# Nucleoid occlusion protein Noc recruits DNA to the bacterial cell membrane

**DOI:** 10.15252/embj.201490177

**Published:** 2015-01-07

**Authors:** David William Adams, Ling Juan Wu, Jeff Errington

**Affiliations:** Centre for Bacterial Cell Biology, Medical School, Newcastle UniversityNewcastle Upon Tyne, UK

**Keywords:** *Bacillus subtilis*, FtsZ, Noc, nucleoid occlusion, ParB

## Abstract

To proliferate efficiently, cells must co-ordinate division with chromosome segregation. In *Bacillus subtilis,* the nucleoid occlusion protein Noc binds to specific DNA sequences (NBSs) scattered around the chromosome and helps to protect genomic integrity by coupling the initiation of division to the progression of chromosome replication and segregation. However, how it inhibits division has remained unclear. Here, we demonstrate that Noc associates with the cell membrane via an N-terminal amphipathic helix, which is necessary for function. Importantly, the membrane-binding affinity of this helix is weak and requires the assembly of nucleoprotein complexes, thus establishing a mechanism for DNA-dependent activation of Noc. Furthermore, division inhibition by Noc requires recruitment of NBS DNA to the cell membrane and is dependent on its ability to bind DNA and membrane simultaneously. Indeed, Noc production in a heterologous system is sufficient for recruitment of chromosomal DNA to the membrane. Our results suggest a simple model in which the formation of large membrane-associated nucleoprotein complexes physically occludes assembly of the division machinery.

## Introduction

Division site selection is a widespread biological problem. Cell division must be spatially and temporally regulated to ensure that progeny are suitably sized and that each receives an intact copy of the genome. Geometry-sensing mechanisms that link division to cell-size or chromosome segregation appear to represent a convenient solution to this problem and are found in single-celled organisms from bacteria to yeast (Moseley & Nurse, 2010). In the majority of bacteria, division is initiated by assembly of the tubulin homologue FtsZ into a membrane-tethered ring-like structure, the Z-ring (Bi & Lutkenhaus, 1991), which serves as a dynamic framework for assembly of the cytokinetic machinery (Adams & Errington, 2009; de Boer, 2010; Erickson *et al*, 2010; Lutkenhaus *et al*, 2012). In the rod-shaped bacteria *Bacillus subtilis* and *Escherichia coli*, which represent the best-studied Gram-positive and Gram-negative model organisms, division site selection is primarily controlled by two negative regulatory systems, Min and nucleoid occlusion. These systems act by using the cell poles and the nucleoid (bacterial “chromosome”), respectively, as geometric cues. Thus, the Min system prevents division at the poles (de Boer *et al*, 1989; Lutkenhaus, 2007) and nucleoid occlusion prevents division over the DNA (Wu & Errington, 2012). The combined action of these two overlapping systems helps to ensure that Z-ring assembly only occurs efficiently at mid-cell (Rodrigues & Harry, 2012).

The ability of the nucleoid to influence division site selection has long been recognised, and various models have been proposed to explain this activity (Mulder & Woldringh, 1989; Woldringh *et al*, 1990, 1991; Sun & Margolin, 2004). However, it was only in the last decade that factors specifically involved in nucleoid occlusion were identified; Noc in *B. subtilis* and SlmA in *E. coli* (Wu & Errington, 2004; Bernhardt & de Boer, 2005). Noc is a ParB homologue that appears to have originated by a partial gene duplication involving *spo0J* (Wu & Errington, 2004). SlmA is a member of the unrelated tetracycline repressor (TetR) family of DNA-binding proteins and is thought to act by interacting directly with FtsZ to inhibit or otherwise perturb its assembly (Bernhardt & de Boer, 2005; Cho *et al*, 2011; Tonthat *et al*, 2011, 2013; Cho & Bernhardt, 2013; Du & Lutkenhaus, 2014). Though neither gene is normally essential in their respective organisms, both are synthetic lethal with mutations in *min*. Simultaneous inactivation of both systems leads to chaotic FtsZ assembly such that it cannot reach a sufficiently high concentration for Z-ring assembly at any one point in the cell, rendering cells unable to divide (Wu & Errington, 2004; Bernhardt & de Boer, 2005). Nevertheless, under conditions that perturb DNA replication, the absence of *noc* (or *slmA* in *E. coli*) is itself sufficient to allow division through the nucleoid (Bernhardt & de Boer, 2005; Wu *et al*, 2009). More recently, Veiga *et al* reported that a *noc* deletion in *Staphylococcus aureus* (which lacks Min) led to Z-ring assembly over the nucleoid and resulted in irreparable DNA damage, thus highlighting a critical role for nucleoid occlusion in this important human pathogen (Veiga *et al*, 2011).

We previously showed that Noc is a sequence-specific DNA-binding protein (Wu *et al*, 2009). It is also an abundant protein, with around 4,500–7,500 molecules per cell (Wu *et al*, 2009; Muntel *et al*, 2014). Indeed, ChAP-chip experiments showed that it forms nucleoprotein complexes at about 70 discrete palindromic Noc-binding sites (NBSs), which are distributed around most of the chromosome (Wu *et al*, 2009). Significantly, the absence of NBSs from the terminus region enables Noc to act as a timing device by allowing division to be initiated a little before chromosome replication and segregation draw to a close. A similar distribution of SlmA-binding sequences (SBSs) has been identified in *E. coli* (Cho *et al*, 2011; Tonthat *et al*, 2011). Importantly, the ability of both Noc and SlmA to inhibit cell division is enhanced by their respective binding sequences (Wu *et al*, 2009; Cho *et al*, 2011; Tonthat *et al*, 2011). Consistent with its role in preventing division over the nucleoid, Noc overproduction causes a mild division block and strongly blocks the initiation of sporulation by preventing assembly of the asymmetric septum (Sievers *et al*, 2002; Wu & Errington, 2004).

Despite its importance, a detailed molecular understanding of the mode of action of Noc has been hindered by the lack of an identified target in the division machinery (Wu *et al*, 2009; Wu & Errington, 2012). To gain fresh insights into how Noc mediates nucleoid occlusion, we have investigated the requirements for Noc activity in *B. subtilis*. Our results demonstrate that unusually, Noc is a DNA-dependent membrane-binding protein that associates with the cell membrane directly via a highly conserved N-terminal motif. Furthermore, we show that simultaneous binding to DNA and the membrane is necessary for Noc function, providing evidence that the mechanism by which Noc acts requires recruitment of DNA to the bacterial cell membrane.

## Results

### Noc localisation is sensitive to membrane potential

**Figure 1:**
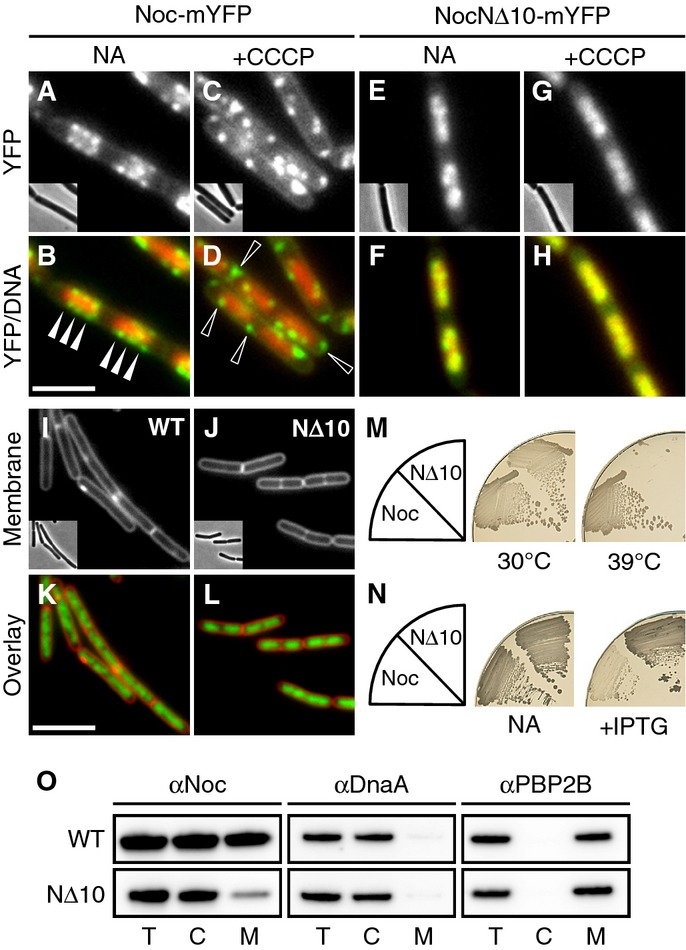
Noc associates with the membrane in a ΔΨ-sensitive manner

We previously reported that Noc forms dynamic foci at the cell periphery (Wu *et al*, 2009). These foci are not an artefact of fluorescent protein-mediated clustering (Landgraf *et al*, 2012) as a monomeric YFP fusion has an indistinguishable localisation pattern (Fig1A and B). TIRF microscopy revealed that the Noc foci move rapidly at the cell surface above the nucleoids, although other than this constraint their movement did not follow any obvious pattern (Supplementary Movie S1). Since the divisome is a membrane-associated complex, we hypothesised that the peripheral foci might represent sites of interaction between Noc and its target, for example FtsZ or one of a dozen or so other division proteins. However, despite extensive analysis, we found no evidence to support a direct interaction between Noc and FtsZ or any other known division protein (Wu *et al*, 2009; Adams *et al*, 2011; Wu & Errington, 2012).

Effect of CCCP on the localisation of Noc and NocNΔ10. Cellular localisation of Noc-mYFP (DWA206) and NocNΔ10-mYFP (DWA382) either with no additions (NA) or after CCCP treatment (5 min; 100 μM), as indicated. Scale bar, 2.5 μm.

Effect of Noc overproduction on cell division. Exponentially growing cultures of DWA119 (Δ*noc*,*P*_spac(hy)-_*noc*) and DWA282 (Δ*noc*,*P*_spac(hy)-_*noc*NΔ10) were examined after growth for 1 h with 1 mM IPTG. Cell membranes and DNA were stained with FM5-95 and DAPI, respectively. Insets show corresponding phase contrast images. Scale bar, 5 μm.

Complementation of *noc* in a Δ*noc* Δ*minCD* background. Strains DWA564 (Δ*noc,* Δ*minCD, P*_*xyl*_-*noc-myfp*) and 566 (Δ*noc,* Δ*minCD, P*_*xyl*_-*noc*NΔ10*-myfp*) were streaked on nutrient agar (NA) plates in the presence of 0.5% w/v xylose and incubated at 30 and 39°C, as indicated.

Effect of Noc and NocNΔ10 overproduction on sporulation. Strains DWA119 (Δ*noc*,*P*_spac(hy)_-*noc*) and DWA282 (Δ*noc*,*P*_spac(hy)_-*noc*NΔ10) were streaked on NA plates in the absence and presence of 1 mM IPTG, as indicated, and photographed after 48 h at 37°C.

Western blot analysis of cellular fractions (T, total; C, cytosolic; M, membrane) of strains DWA119 and 282 expressing Noc or NocNΔ10, respectively. Proteins were detected using polyclonal antibodies against Noc, DnaA and PBP2B. Antibodies were used at a dilution of 1:10,000.

The transmembrane electrical potential (ΔΨ) was recently shown to influence the localisation of a variety of peripheral and integral membrane proteins involved in cell morphogenesis and division (Strahl & Hamoen, 2010). To test whether ΔΨ plays a role in the peripheral localisation of Noc, we examined cells treated with the proton ionophore, carbonyl cyanide *m*-chlorophenyl hydrazone (CCCP), which dissipates both ΔΨ and the transmembrane chemical proton gradient (ΔpH). Strikingly, Noc localisation was radically altered within a few minutes of CCCP treatment (Fig1C and D). Although the protein still formed multiple peripheral foci, these were no longer restricted to the nucleoid and, in particular, some foci were now apparent unusually close to the cell poles (compare arrowheads in Fig1B and D). Importantly, TIRF microscopy revealed that although these foci were still present at the cell surface, they were virtually static (Supplementary Movie S2). Nigericin, which dissipates the ΔpH only, showed no effect on Noc localisation (compare Supplementary Fig S1A and B). Furthermore, CCCP-dependent delocalisation of Noc also occurred in an F_1_F_0_ ATP synthase deletion background (Supplementary Fig S1C and D). These results suggest that Noc delocalisation is due specifically to the loss of ΔΨ and is not an indirect consequence of ATP depletion. We therefore conclude that much of the cellular complement of Noc localises in rapidly moving foci that are sensitive to the ΔΨ.

### Noc contains a highly conserved N-terminal motif predicted to form an amphipathic helix

**Figure 2:**
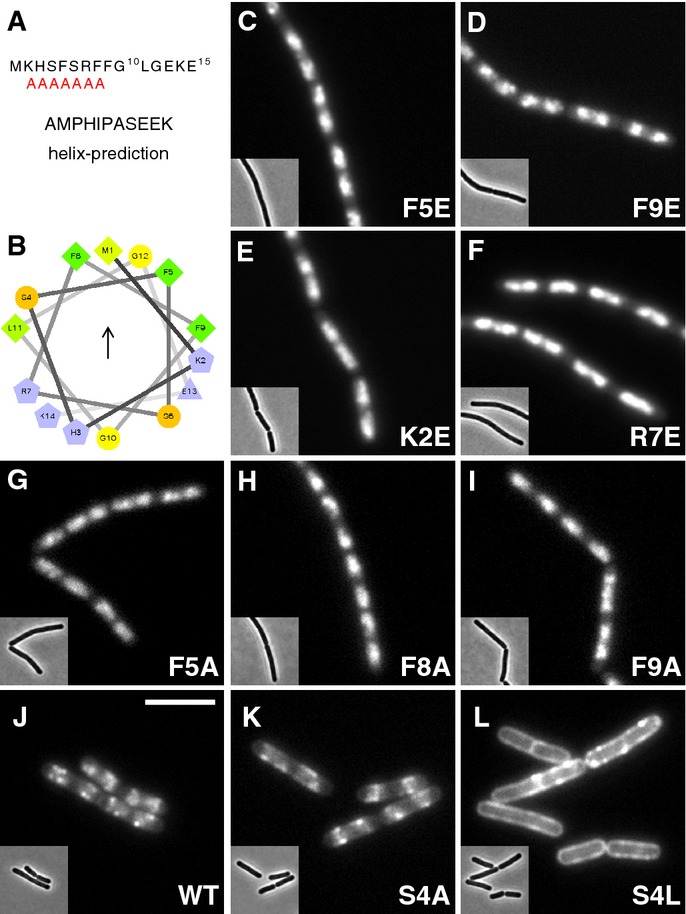
The N-terminus of Noc contains an amphipathic helix

The CCCP sensitivity of MinD localisation was shown to be a result of the ΔΨ-stimulated membrane binding of its amphipathic helix (Strahl & Hamoen, 2010). Analysis of Noc using AMPHIPASEEK, a program designed to look for amphipathic in-plane membrane anchors (Sapay *et al*, 2006), highlighted a possible N-terminal amphipathic helix (Fig2A). A helical wheel projection of the N-terminus (aa 1–14) broadly conforms to the canonical organisation of an amphipathic helix, with a hydrophobic face opposed by a more polar one and with positively charged residues flanking the hydrophobic face (Fig2B). In support of the idea that this region plays an important role in Noc function, multiple sequence alignments of Noc proteins from a range of organisms revealed an extremely high level of conservation in the extreme N-terminus (ca. aa 1–15) followed by a short stretch of much higher variability, which we speculate may represent a variable spacer (Supplementary Fig S2A and B). AMPHIPASEEK scans of all Noc homologues examined all predicted an N-terminal amphipathic helix (not shown). Interestingly, the N-terminus of Spo0J (ParB) mediates the interaction with its partner, Soj (ParA) (Gruber & Errington, 2009; Scholefield *et al*, 2011). As expected, given their paralogous nature, Noc and Spo0J share extensive regions of homology. However, consistent with the idea that they have evolved to perform disparate roles, the N-termini of the two proteins are clearly divergent (Supplementary Fig S2B) and Spo0J localisation is not sensitive to ΔΨ (Strahl & Hamoen, 2010).

AMPHIPASEEK prediction result for *Bacillus subtilis* Noc. The red “A”s indicate a putative amphipathic helical region.

Helical wheel projection of the N-terminus (aa 1–14) showing the presence of hydrophobic (arrow) and polar faces. Residues are coloured according to their properties, greens, hydrophobic; blues, charged; orange, polar, uncharged; and yellow, glycine. The figure was prepared using the tool available at http://rzlab.ucr.edu/scripts/wheel/wheel.cgi.

Effects of N-terminal substitutions on Noc localisation, in strains: DWA211 (F5E), 318 (F9E), 316 (K2E), 212 (R7E), 322 (F5A), 323 (F8A), 325 (F9A), 206 (WT), 328 (S4A) and 329 (S4L), as indicated. Insets show the corresponding phase contrast images. Scale bar, 5 μm.

### The N-terminus of Noc is required for membrane localisation and protein function

To test directly whether the N-terminus is required for the peripheral localisation of Noc, we constructed an N-terminally truncated Noc variant lacking the first 10 amino acids (NocNΔ10). NocNΔ10 retained the ability to localise to the nucleoid, but it appeared not to form the peripheral foci characteristic of the wild-type protein (Fig1E and F and Supplementary Movie S3). Crucially, CCCP treatment had no effect on the localisation of NocNΔ10 (Fig1G and H) consistent with the N-terminus of the protein mediating the ΔΨ-sensitive interaction with the cell periphery. Moreover, the truncated protein was not functional as it did not rescue the synthetic division defect of a *noc minCD* double mutant that arises at temperatures ≥ 37°C (Fig1M), and when overproduced, it did not inhibit division (Fig1I–L and Supplementary Fig S4A) or sporulation (Fig1N; compare dense Spo^+^ and pale Spo^−^ colonies). To test more directly for a Noc–membrane interaction, we examined whether Noc could be detected in purified membrane preparations using an integral membrane protein (PBP2B) and an unrelated DNA-binding protein (DnaA) as fractionation controls. In contrast to the well-characterised DNA-binding protein, DnaA, which is found almost exclusively in the cytosol, almost half of the wild-type Noc appeared in the membrane fraction (Fig 1O). Although a trace of NocNΔ10 was detected in the membrane fraction, the vast majority of the protein was cytosolic (Fig 1O). Additionally, size-exclusion chromatography of purified NocNΔ10 confirmed that it is properly folded and, as for the full-length protein, forms multimers in solution (Supplementary Fig S3).

Amphipathic helices bind to the membrane by inserting their hydrophobic face into the bilayer and are often stabilised by electrostatic interactions between positively charged residues and the negatively charged polar lipid head-groups (Cornell & Taneva, 2006). To test whether the N-terminus mediates membrane binding directly, we made mutations predicted to affect the key properties of the putative amphipathic helix and tested their effects on localisation and function. Importantly, introducing negative charges (i.e. F5E or F9E) into the predicted hydrophobic face of the helix (Fig2C and D) or inverting the flanking positive charges (i.e. K2E or R7E) (Fig2E and F), all abolished peripheral but not nuclear localisation. Furthermore, both types of substitutions led to loss of protein function (Supplementary Table S1 and Supplementary Fig S4A and B). Similarly, making deletions that disrupt the amphipathic organisation of the helix, that is ΔK2 or ΔF5, S6 also rendered the protein partially functional (Supplementary Fig S4B).

The results above suggested that the positive charges flanking the hydrophobic face of the predicted helix sit in proximity to the membrane. However, further analysis showed that individually, these charges (i.e. K2, R7 and K14) are dispensable for Noc activity (Supplementary Fig S4B), which suggested that membrane binding could be driven principally by the hydrophobic face itself. Indeed, reducing the hydrophobicity of the predicted membrane-binding face by individually replacing any one of the three phenylalanines present with alanine (i.e. F5A, F8A or F9A) resulted in the loss of peripheral foci and the loss of protein function (Fig2G–I and Supplementary Table S1). Conversely, increasing the hydrophobicity of this region by introducing either alanine or leucine in place of the serine at position 4 led to enhanced membrane localisation. In the case of S4L, this effect was dramatic (compare Fig2J–L) and also led to enhanced protein function, as evidenced by the ability of this variant to rescue the growth of a *noc minCD* double mutant at lower levels of induction than the WT (Supplementary Table S1). Interestingly, multiple sequence alignments revealed a clear preference for a non-canonical residue (e.g. S, T or P) at this position, which we hypothesise might act to regulate or otherwise limit the membrane-binding affinity of the helix (see Discussion).

### A heterologous amphipathic helix can functionally substitute for the N-terminus

**Figure 3:**
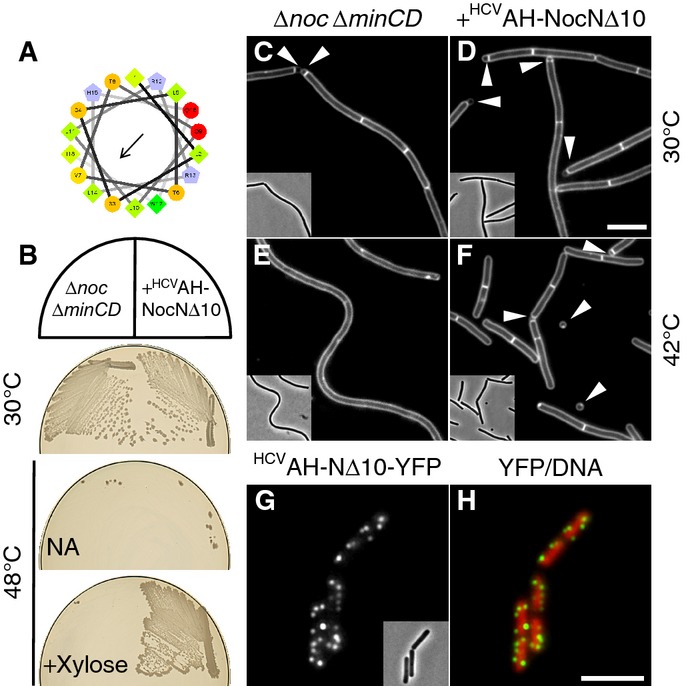
A heterologous amphipathic helix can functionally substitute for the N-terminus

The above data are consistent with a model in which Noc interacts with the membrane directly via an N-terminal amphipathic helix. If correct, the addition of a heterologous membrane-targeting sequence should restore activity to the otherwise non-functional NocNΔ10 truncation. We therefore constructed a chimaeric fusion between the well-characterised amphipathic helix from the hepatitis C virus protein, NS4B (^HCV^AH) (Fig3A) (Gouttenoire *et al*, 2009), and NocNΔ10, connected by a short flexible linker (^HCV^AH-NocNΔ10). To test whether the chimaeric protein was functional, we introduced it into the temperature-sensitive Δ*noc* Δ*minCD* background. As expected, although both strains could grow and divide reasonably efficiently at 30°C (Fig3B–D), the parental strain became highly filamentous at 42°C (Fig3E) and could not grow at 48°C under any of the conditions examined (Fig3B). In contrast, the strain carrying the chimaeric gene was able to grow at 48°C when its expression was induced (addition of ≥ 0.05% xylose) (Fig3B). Microscopic examination of the cells showed that the growth restoration was associated with an enhanced rate of division and the frequent formation of minicells (Fig3F), which are characteristic of a Min^−^ single mutant phenotype. Expression of the same amphipathic helix fused to GFP had no functionality (Supplementary Fig S5A).

Helical wheel projection showing the amphipathic helix from hepatitis C virus protein NS4B. The hydrophobic face is indicated by an arrow. Colour scheme as in Fig2B.

Growth of strains DWA350 (Δ*noc* Δ*minCD*) and DWA307 (Δ*noc* Δ*minCD*,*P*_*xyl*_-^HCV^AH-NocNΔ10) on nutrient agar plates at 30 and 48°C in the absence and presence of 0.5% w/v xylose, as indicated.

Cell morphology of strains DWA350 and DWA307 following growth in LB at 30°C (C and D) and at 42°C in LB + 0.5% w/v xylose (E and F). Arrowheads indicate minicells. Cell membranes were stained with FM5–95. Insets show the corresponding phase contrast images. Scale bar, 5 μm.

Cellular localisation of ^HCV^AH-NocNΔ10-YFP (G) in strain DWA193 (Δ*noc*,*P*_*xyl*_-^HCV^AH-*noc*NΔ10-*yfp*) and overlay showing DAPI-stained DNA (H). The strain was grown at 30°C in CH medium. Inset shows the corresponding phase contrast image. Scale bar, 5 μm.

To test whether the addition of the HCV amphipathic helix was sufficient to restore the peripheral localisation of NocNΔ10, we created a C-terminal YFP fusion (^HCV^AH-NocNΔ10-YFP) and determined its cellular localisation. The chimaera formed discrete foci scattered at the cell periphery over the nucleoids (Fig3G and H), and although their distribution was somewhat different from that of the wild-type protein, possibly because of its higher membrane affinity, the fusion protein was also functional (Supplementary Fig S5B). Thus, the N-terminus can be replaced by a completely heterologous amphipathic helix, consistent with the idea that its principal role is to act as a membrane-targeting sequence. In contrast, although the addition of a synthetic transmembrane (TM) domain [WALP23; (Nyholm *et al*, 2007)] led to stable association of NocNΔ10 with the cell membrane, it was unable to rescue the growth of a *noc min* mutant (Supplementary Fig S5C and D). Moreover, expression of the TM-NocNΔ10 variant led to defects in chromosome segregation, with clear evidence of broken and even bisected chromosomes, in many of the cells (Supplementary Fig S5D).

### Membrane binding of Noc requires spreading on DNA

Surprisingly, Noc appears to have only weak affinity for the cell membrane, as fusions between the N-terminal peptide and GFP, either with or without an artificial dimerisation domain, did not target GFP to the membrane (Supplementary Fig S6). Similarly, a non-DNA-binding Noc variant (K164A) (Wu *et al*, 2009) localised diffusely in the cytoplasm and was non-functional (Supplementary Table S1). Previously, we showed that Noc, like its relative Spo0J and other ParB-family proteins, forms large nucleoprotein complexes on DNA via an activity termed “spreading” (Rodionov *et al*, 1999; Murray *et al*, 2006; Breier & Grossman, 2007; Wu *et al*, 2009). Briefly, a primary (C-terminal) dimerisation domain facilitates initial dimerisation and DNA binding. Once bound to DNA, however, a secondary dimerisation domain maintains the dimer, enabling the protein to oligomerise via its C-terminal domain and thus “spread” outwards on adjacent non-specific DNA (Leonard *et al*, 2004). Recent work suggests that in addition to these nearest-neighbour interactions, spreading may also proceed by bridging loops of DNA (Graham *et al*, 2014). We hypothesised that spreading might compensate for the weak binding affinity of individual N-termini by concentrating them in large complexes. Indeed, there are sufficient Noc molecules to form an oligomer of around 1–2 kb per NBS (Wu *et al*, 2009).

**Figure 4:**
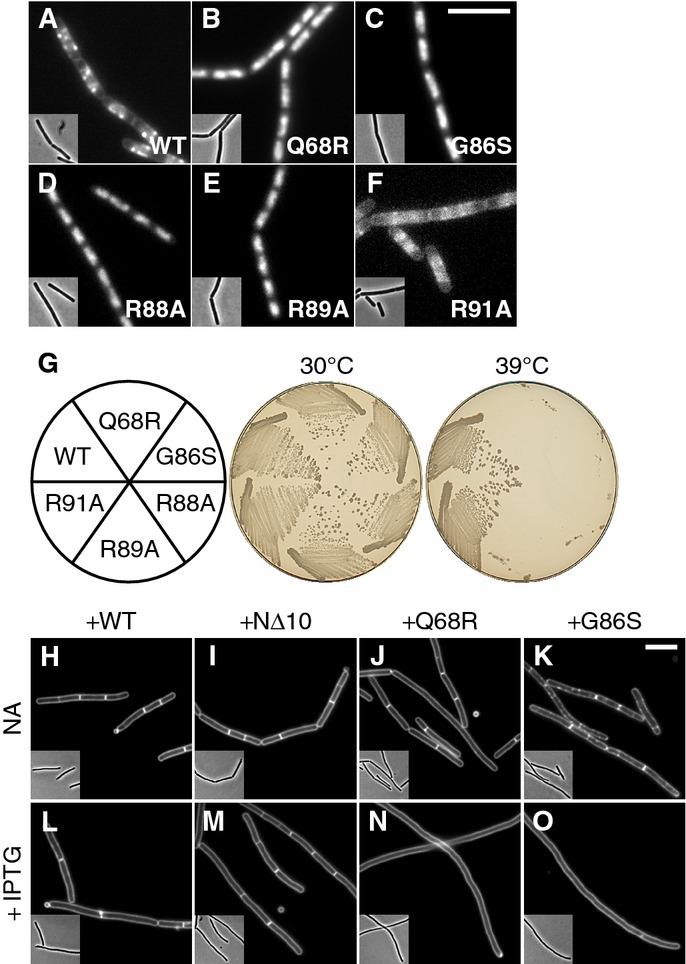
Noc–membrane association requires spreading on DNA

To test this idea, we investigated a series of substitutions within the two highly conserved ParB-boxes (Yamaichi & Niki, 2000) (Supplementary Fig S7A), which sit together in the *Thermus thermophilus* Spo0J crystal structure and are thought to control spreading by forming the secondary dimerisation domain (Leonard *et al*, 2004). Well-characterised variants of *B. subtilis* Spo0J with substitutions at G77S, R79A or R80A are known to be defective in spreading, but not DNA binding (Breier & Grossman, 2007; Graham *et al*, 2014). A related variant, R82A, is still able to spread but has diminished activity (Graham *et al*, 2014). We therefore constructed four *noc* alleles encoding the equivalent substitutions (i.e. G86S, R88A, R89A and R91A) as well as an allele encoding Q68R within ParB-box I, which was isolated during the course of this work (D. W. Adams & J. Errington, unpublished observations) and tested their functionality. All of the residues examined are universally conserved between Noc and Spo0J homologues (Supplementary Fig S7A). Significantly, each of the substitutions, except R91A, abolished the ability of Noc to form foci at the cell periphery, although they were still clearly nucleoid associated (compare Fig4A with B–E). Like other non-membrane-binding mutants, they were unable to complement the growth defect of a Δ*noc* Δ*minCD* strain (Fig4G) and they did not inhibit sporulation when overproduced (not shown). R91A, on the other hand, showed a weakened WT localisation pattern (Fig4F), consistent with the partially functional phenotype of its Spo0J equivalent (Graham *et al*, 2014). To verify that these mutants are defective in complex formation, we took advantage of the fact that the NBSs (and thus, normally, Noc) are largely absent in the terminus region of the chromosome (Wu *et al*, 2009). When the terminus region was labelled using a fluorescent reporter operator system (FROS), the ParB-box mutants frequently overlapped with *terC* (Q68R, 93%, *n *=* *114; G86S, 94%, *n *=* *117), whereas the WT (39%, *n *=* *212) and NΔ10 (40%, *n *=* *139) proteins did so much less frequently, supporting the idea that the ParB-box mutants bind non-specifically over the entire chromosome. Further highlighting the important role of the ParB-boxes, even the S4L-enhanced membrane-binding variant of Noc no longer associated with the membrane and was rendered non-functional when combined with the G86S substitution (Supplementary Table S1).

Localisation of ParB-box mutants. Cellular localisation of (A) Noc-mYFP (DWA206), (B) NocQ68R-mYFP (DWA285), (C) NocG86S-mYFP (DWA286), (D) NocR88A-mYFP (DWA545), (E) NocR89A-mYFP (DWA546) and (F) NocR91A-mYFP (DWA547). Insets show the corresponding phase contrast images. Scale bar, 5 μm.

Ability of ParB-box mutants to rescue the growth defect of Δ*noc* Δ*minCD*. Strains DWA564 (*P*_*xyl*_-*noc-myfp*), 590 (*P*_*xyl*_-*noc*Q68R*-myfp*), 568 (*P*_*xyl*_-*noc*G86S*-myfp*), 598 (*P*_*xyl*_-*noc*R88A*-myfp*), 600 (*P*_*xyl*_-*noc*R89A*-myfp*) and 602 (*P*_*xyl*_-*noc*R91A*-myfp*) were streaked on plates containing 0.5% w/v xylose and incubated for 18 h at either 30 or 39°C, as indicated, before being photographed.

ParB-box mutants are dominant-negative. Cells of strains DWA362 (Δ*minCD*,*P*_spac(hy)-_*noc*), 363 (Δ*minCD*,*P*_spac(hy)-_*noc*NΔ10), 364 (Δ*minCD*,*P*_spac(hy)-_*noc*Q68R) and 365 (Δ*minCD*,*P*_spac(hy)-_*noc*G86S) were examined after growth for 2 h at 42°C with either no additions (NA) (H–K) or in the presence of 1 mM IPTG (L–O), as indicated. Cell membranes were stained with FM5-95. Insets show the corresponding phase contrast images. Scale bar, 5 μm.

### ParB-box mutants are dominant-negative

The phenotype of the spreading-defective mutants was superficially similar to that of the NΔ10 truncation. However, they might be expected to behave in different ways when co-expressed with wild-type *noc*. We hypothesised that if hetero-dimers of native Noc and the ParB-box mutants were also defective in complex formation, then these mutants might have a dominant-negative phenotype. To test this prediction, we overproduced various forms of Noc in a *noc*^+^ Δ*minCD* background and incubated the cultures at 42°C. In the absence of inducer, all strains were able to divide efficiently (Fig4H–K). Following induction, however, whereas the strains overproducing either Noc or NocNΔ10 continued to divide (Fig4L and M), overproduction of the ParB-box mutants produced a severe division defect, leading to the formation of long aseptate filaments (Fig4N and O). Similarly, *noc*^+^ Δ*minCD* strains overproducing the non-functional ParB-box mutants (i.e. Q68R, G86S, R88A or R89A) failed to grow on plates at the restrictive temperature, whereas overproduction of Noc, NocNΔ10 or the spreading-impaired R91A mutant did not (Supplementary Fig S7B and C). Consistent with these findings and the idea that the ParB-box mutants cannot form productive complexes, overproduction of Noc restored the peripheral localisation of NocNΔ10, but not NocG86S (Supplementary Fig S7D–G).

### Noc recruits NBS DNA to the cell membrane

**Figure 5:**
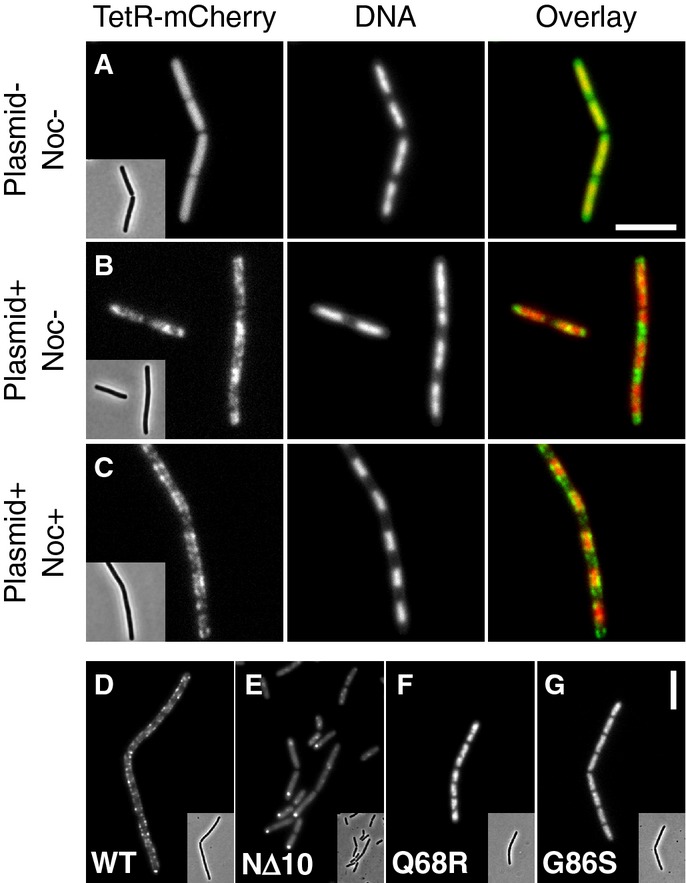
Noc recruits NBS DNA to the cell membrane

We previously showed that the presence of a NBS sequence on a multi-copy plasmid generates a severe Noc-dependent division phenotype (Wu *et al*, 2009). In the light of the above results, we anticipated that the division block results from the recruitment of Noc-plasmid complexes to the cell membrane: since plasmid localisation is less constrained than that of the chromosome, division inhibition occurs throughout the cell, rather than only over the nucleoid. To test this idea, we constructed a multi-copy NBS plasmid that could be labelled using a TetR/*tetO* FROS and used this to examine the effects of Noc on plasmid localisation. As expected, TetR-mCherry was uniformly distributed throughout the cytoplasm of otherwise wild-type cells (Fig5A). When the NBS plasmid was introduced, but in the absence of Noc (the sole copy of *noc* was placed under the control of the *P*_*spac*_ promoter), the mCherry signal localised in the cytoplasm, primarily at the cell poles and in the inter-nucleoid spaces (Fig5B), in line with the reported distribution of similarly sized plasmids in *E. coli* (Reyes-Lamothe *et al*, 2014). When Noc synthesis was induced, the cells exhibited a severe division block (Supplementary Fig S8A–D), similar to that previously described, and this was accompanied by a conspicuous change in plasmid localisation (Fig5C): now they formed foci that were clearly recruited to the cell periphery, where they moved dynamically along the entire length of the cell (compare Fig5B and C and Supplementary Movies S4 and S5).

Representative images of TetR-mCherry in cells lacking (A) or containing (B and C) the NBS plasmid pDWA117. Strains DWA427 (A) and 429 (B and C) (both strains contain *P*_spac_-*noc*) were examined after growth for 2 h in the absence (A and B) and presence (C) of 1 mM IPTG. DNA was stained with DAPI.

Representative images showing the localisation of (D) Noc-mYFP (DWA519), (E) NocNΔ10-mYFP (DWA522), (F) NocQ68R-mYFP (DWA520) and (G) NocG86S-mYFP (DWA521), in the presence of the NBS plasmid pSG4929. Data information: Insets show the corresponding phase contrast images. Scale bars, 5 ?m.

The apparent distribution of plasmids in the presence of Noc was very similar to that of Noc in the presence of an NBS plasmid (Wu *et al*, 2009) (Fig5D), consistent with the protein and plasmid DNA forming a co-complex. Notably, neither class of non-functional mutants (i.e. NocNΔ10 or Q68R, G86S, R88A and R89A) exhibited an association with the membrane in this system nor did they inhibit division, even when overproduced (Fig5E–G and Supplementary Fig S8E). However, they had very different localisation patterns. The NΔ10 truncation exhibited weak nucleoid-like staining and formed prominent cytoplasmic foci, consistent with it being diluted away from the nucleoid by the plasmids (Fig5E). We assume that this reflects the higher total copy number of NBSs on the multi-copy plasmid. In contrast, the ParB-box mutants (Q68R and G86S) had a strong nucleoid-like localisation pattern (Fig5F and G), in agreement with an ability to bind to DNA, but not to form complexes at NBSs. Indeed, since chromosomal DNA far exceeds plasmid DNA in mass this pattern is not unexpected.

### Noc recruits DNA to the membrane in a heterologous system

**Figure 6:**
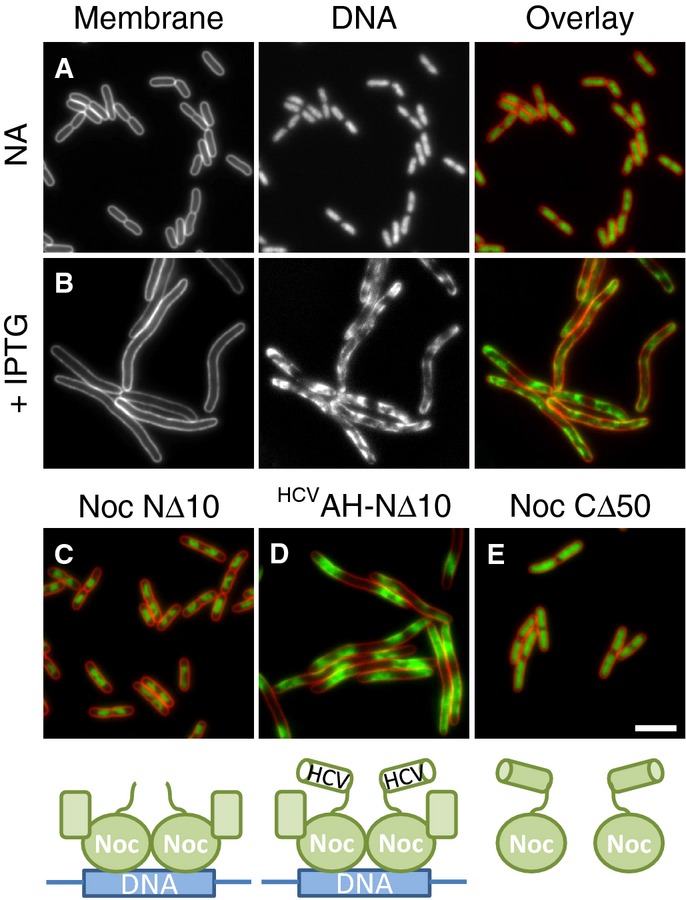
Noc overproduction recruits chromosomal DNA to the membrane in *Escherichia coli*

The results presented above suggest a model whereby Noc acts directly to recruit DNA to the bacterial cell membrane. Such a mechanism might be expected to work in a heterologous system. Therefore, as a final test of this model, we expressed *noc* from a high copy number plasmid in the distantly related Gram-negative bacterium *E. coli*. Un-induced cells grew normally and contained nucleoids that were indistinguishable from the empty vector control (Fig6A). In contrast, induced cells contained nucleoids with a drastically altered conformation, such that the DNA now occupied the majority of the cell periphery (Fig6B). To test whether this effect was the direct result of Noc recruiting chromosomal DNA to the inner membrane, we examined whether the various *noc* alleles generated to test Noc function in *B. subtilis* behaved similarly in the heterologous system. Indeed, removal of the N-terminal 10 amino acids of Noc abolished this effect, but could be reinstated by adding back the heterologous ^HCV^AH (compare Fig6C and D). Additionally, overproduction of a C-terminally truncated Noc (CΔ50), which prevents dimerisation and thus DNA binding, had no effect on nucleoid appearance (Fig6E and Supplementary Fig S3). Since Western blotting indicated that all the variants are produced at similar levels (Supplementary Fig S9A), these results support the idea that this effect is specific.

Effects of Noc overproduction on cell division and nucleoid morphology in *E. coli*. Cells of strain DWA261 carrying pDWA37 (*P*_A__1/04/03_-*noc*) were examined after growth in LB with either no additions (A) or after induction for 1 h with 1 mM IPTG (B).

Effects of overproduction of Noc variants on nucleoid morphology. Cells of strains DWA266 (*P*_A__1/04/03_-*noc*NΔ10) (C), 270 (*P*_A__1/04/03_-^HCV^AH-*noc*NΔ10) (D) and 267 (*P*_A__1/04/03_-*noc*CΔ50) (E) carrying plasmids for the overproduction of the indicated mutants (see cartoons underneath panels) were grown in LB and examined after growth for 1 h in the presence of 1 mM IPTG. Cell membranes and DNA were stained with FM5-95 and DAPI, respectively. Scale bar, 5 μm.

**Figure 7:**
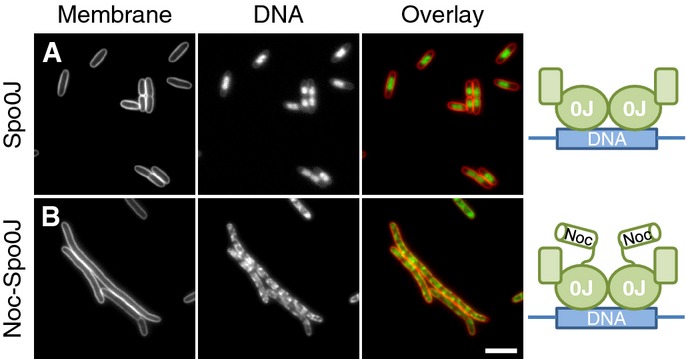
A Noc-Spo0J hybrid can recruit DNA to the membrane in *E. coli*

These results are consistent with Noc binding simultaneously to DNA and the cell membrane without the need of a specific protein partner. If correct, we reasoned it might be possible to reconstitute this process by combining the membrane-targeting activity of the Noc N-terminus with the DNA-binding activity of its paralogue Spo0J. To test this, we constructed a Noc-Spo0J hybrid by fusing the N-terminal 30 amino acids of Noc directly onto Spo0J. Significantly, when overproduced at similar levels (Supplementary Fig S9B), the Noc-Spo0J hybrid, but not Spo0J itself, recruited DNA to the cell periphery (compare Fig7A and B). This result shows that the N-terminus of Noc is a transplantable membrane-targeting sequence, and strongly supports the hypothesis that its activity is coupled to DNA binding.

Effects of Spo0J and Noc30-Spo0J overproduction on cell division and nucleoid morphology in *E. coli*. Cells of strains DWA271 (*P*_A__1/04/03_-*spo0J*) (A) and 272 (*P*_A__1/04/03_-*noc*-*spo0J*) (B) were grown in LB in the presence of 1 mM IPTG, to induce the expression of either Spo0J (A) or the Noc-Spo0J hybrid (B), and were examined 1 h post-induction. Cell membranes and DNA were stained with FM5–95 and DAPI, respectively. Scale bar, 5 μm.

Finally, we noted that the recruitment of DNA to the membrane was accompanied by a clear block in cell division (Figs6B and 7B). This block was independent of known division inhibitors MinCD, SlmA and SulA, indicating that the effect is probably not a secondary consequence of activating an *E. coli* division inhibitor (Supplementary Fig S10). However, since *E. coli* lacks any consensus NBSs, it is likely that Noc–DNA binding occurs either in a largely un-restricted manner over the entire chromosome or else at some of the near-consensus sequences present (68 sites; 2 bp mismatch). We are therefore unable to exclude the possibility that the gross alterations of chromosome structure induced by Noc have an indirect effect on division by affecting critical but unrelated cellular processes.

## Discussion

### Noc is a peripheral membrane protein

Multiple lines of evidence suggest that the extreme N-terminus of Noc mediates membrane binding by forming an amphipathic helix. Removal of the N-terminal 10 amino acids led to loss of membrane association, loss of ΔΨ-sensitivity and, importantly, loss of protein function. Disrupting the predicted membrane-binding face of the helix, by either introducing negative charges or reducing its hydrophobicity, led to loss of membrane association, whereas mutations that increased the hydrophobic nature of this region led to enhanced membrane association. Consistent with the idea that N-terminus of Noc functions as a membrane-targeting motif, we showed that it can be replaced by a completely unrelated viral amphipathic helix. Taken together, our results show that Noc is a ΔΨ-sensitive peripheral membrane protein that associates with the cell periphery directly via a highly conserved N-terminal motif. The ΔΨ-sensitivity of Noc localisation could result directly from its amphipathic helix binding in a ΔΨ-sensitive manner (Strahl & Hamoen, 2010). Alternatively, the regions of increased membrane fluidity that arise in the absence of ΔΨ may also play a role (Strahl *et al*, 2014).

### Membrane association requires the formation of nucleoprotein complexes

A second class of *noc* mutants revealed that nucleoprotein complex formation is necessary for Noc function. A series of mutations within either of the two highly conserved ParB-boxes (I & II) (Yamaichi & Niki, 2000) present in Noc caused loss of membrane binding and loss of protein function. Mutations within these boxes have previously been shown to diminish or abolish the ability of other ParB homologues, for example Spo0J, to “spread” on DNA (Breier & Grossman, 2007; Kusiak *et al*, 2011). Given the shared ancestry between Noc and Spo0J, the simplest interpretation of the results is that these mutations act via the same mechanism. Indeed, Graham *et al* (2014) recently showed that substitutions in ParB-box II that abolished Spo0J spreading on DNA (i.e. G77S, R79A and R80A) were unable to form the normal Spo0J focus at the chromosomal origin of replication, whereas a partially functional variant (R82A) retained the ability to form weakened foci (Graham *et al*, 2014). Importantly, the behaviour of the equivalent Noc ParB-box mutants precisely mirrors that of the Spo0J substitutions. Moreover, the ParB-box mutants were not excluded from the terminus region, suggesting that they localise over the entire chromosome and they appeared to tend not to bind to plasmids carrying NBSs. Consistent with the idea that this is caused by a perturbation of Noc higher-order assembly, these mutants are dominant-negative, probably because they shorten or terminate the nascent nucleoprotein oligomers.

**Figure 8:**
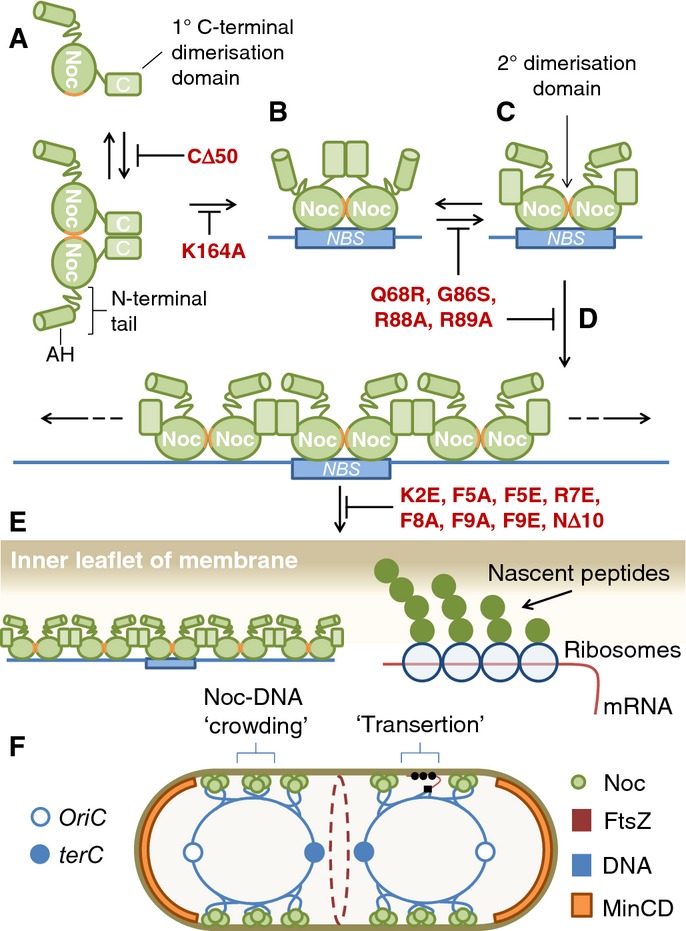
Model for mode of action of Noc Noc dimerises (A) and binds to DNA, nucleating specifically at NBSs (B). DNA-bound dimers are maintained by the secondary dimerisation domain (ParB-boxes, orange) (C) allowing Noc to oliogomerise on adjacent non-specific DNA (D). Nucleoprotein complexes trigger membrane binding by generating clusters of the N-terminal amphipathic helix (E). Membrane-associated Noc complexes are distributed asymmetrically by the NBSs. Crowding by Noc–DNA complexes may physically inhibit division over the nucleoid by biasing FtsZ assembly away from these regions (F). For comparison, the complexes generated by “transertion” are shown alongside. See text for a full description. The schematic depicting Noc complex assembly is adapted from that previously proposed for Spo0J (Leonard *et al*, 2004).

Our finding that the membrane-targeting activity of the N-terminal peptide of Noc is weak and requires the formation of nucleoprotein complexes suggests that oligomerisation is necessary to facilitate membrane binding. Structuring or otherwise concentrating the N-termini in large complexes likely functions to compensate for the weak affinity of the individual termini. The rapid movement of Noc foci that we observed at the cell surface suggests that Noc associates with the membrane in a transient manner, perhaps with fast ON/OFF rates. Stronger modes of binding may have been selected against, as a more stable linkage of chromosome to membrane could be deleterious, for example by hampering gene expression or chromosome replication. A DNA-dependent mode of binding also locks Noc into a pathway by which it first has to bind and spread at NBSs before going to the membrane (Fig8A–D). More stable modes of association would bypass this requirement and by confining Noc to the membrane might limit its ability to bind DNA. Indeed, a TM-Noc variant was non-functional and although it often led to multiple defects in chromosome segregation, the effects were heterogeneous. Another important consequence of coupling membrane binding to nucleoprotein complex formation is that it provides a mechanism for site-specific DNA-dependent activation of Noc and thus explains how the NBSs function to spatially constrain Noc activity.

In *E. coli,* DNA binding at SBSs is thought to stimulate SlmA activity by promoting a conformational change that exposes the FtsZ-binding site (Cho & Bernhardt, 2013). Importantly, however, DNA-binding and SlmA activity appear to be separable since, in contrast to Noc (Wu *et al*, 2009), a non-DNA-binding mutant is capable of inhibiting division, albeit less efficiently (Bernhardt & de Boer, 2005; Cho *et al*, 2011). DNA binding could therefore have a least two functions, (I) to restrict Noc activity to NBSs and (II) to actively participate in the inhibitory mechanism (see below). The results presented in this work clearly show that Noc function requires simultaneous association with both DNA and membrane. This unexpected finding is completely different to that of the only other known nucleoid occlusion protein, SlmA. Indeed, SlmA acts directly on FtsZ polymerisation (Cho *et al*, 2011; Tonthat *et al*, 2011; Du & Lutkenhaus, 2014) and there is no evidence that it associates directly or indirectly with the cell membrane (Cho *et al*, 2011).

### How does Noc inhibit division?

Our data argue against certain potential models for Noc function. First, a direct interaction between Noc and another division protein seems unlikely since extensive analysis using multiple techniques has found no evidence for an interaction partner and the only mutations that affected Noc activity were found in *noc* itself. Furthermore, if the target of Noc were another membrane protein, then placing Noc in continuous proximity to its target by adding a TM domain might be expected to impose a severe division block, which was not the case. Second, as amphipathic helices can perturb the organisation of the membrane (Cornell & Taneva, 2006), Noc might disrupt the association of the proteins (e.g. FtsA or SepF) required for anchoring FtsZ to the membrane (Pichoff & Lutkenhaus, 2005; Duman *et al*, 2013). However, the fact that the N-terminal peptide can be replaced by a totally unrelated viral amphipathic helix and that when overproduced by itself, it has no effect on cell division, argues against a specific role. Moreover, complementation experiments generating mixtures of WT and NocNΔ10 proteins *in vivo* showed that Noc complexes do not need to be saturated with N-termini in order to function.

However, the demonstration that Noc function has a strict requirement for concurrent DNA and membrane association leads us to consider a third model, wherein the delivery of DNA to the cell periphery is itself sufficient to inhibit division (Fig8). Indeed, more than 20 years ago, Woldringh and colleagues proposed that large complexes formed by the coupled transcription–translation–insertion of membrane proteins (“transertion”) might lead to physical crowding over active regions of the nucleoid, enabling it to act as a short-range inhibitor of cell division (Mulder & Woldringh, 1989; Woldringh *et al*, 1990, 1991; Woldringh, 2002). This process would physically bias the division machinery away from regions occupied by these complexes. Though an attractive and widely discussed model, direct evidence for a role in cell division has remained lacking. Given that Noc is able to recruit DNA to the cell membrane and that this process almost certainly involves the formation of large nucleoprotein complexes, a logical hypothesis is that the Noc foci present over the nucleoid represent membrane-associated nucleoprotein complexes. We therefore hypothesise that these large complexes may inhibit division directly by physically crowding the membrane over the nucleoid in a manner similar to that suggested above. Notably, whereas “transertion” generates large complexes of the translational/secretion machineries, crowding generated by Noc would result directly from the recruitment of Noc nucleoprotein complexes and associated DNA to the cell membrane (Fig8E and F). Experiments in *E. coli* showing that simultaneous DNA and membrane association are necessary and sufficient to recruit DNA to the cell periphery are compatible with this model and support the idea that there is no direct target involved in the recruitment process. Therefore, in contrast to other well-characterised regulators of cell division, Noc might not act via a specific protein target. Instead, by facilitating the association of large DNA complexes with the cell periphery, it may simply act to enhance the natural ability of the nucleoid to act as a short-range inhibitor of division.

Although further work will be necessary to define the precise mechanism by which Noc acts, it is clear it does so at the membrane. As such, this work highlights the evolution of a novel mechanism to co-ordinate cell division and chromosome segregation by “repurposing” Spo0J via the acquisition of a membrane-targeting sequence. Finally, many bacteria lack obvious nucleoid occlusion proteins, and in those that do possess them, it is increasingly clear that in even in their absence, the nucleoid or else another associated factor(s) continues mostly to prevent division through the DNA (Wu & Errington, 2004; Bernhardt & de Boer, 2005; Bernard *et al*, 2010; Männik *et al*, 2012; Bailey *et al*, 2014; Cambridge *et al*, 2014). The widespread distribution of ParB-family proteins throughout bacteria raises the exciting possibility that other Noc-like proteins await discovery.

## Materials and Methods

### Bacterial strains and plasmids

The bacterial strains used in this study are shown in Supplementary Table S2, together with the plasmids used and their construction.

### General methods

*Bacillus subtilis* cells were made competent for transformation as previously described (Hamoen *et al*, 2002). DNA manipulations and *E. coli* transformations were carried out using standard methods (Sambrook *et al*, 1989), and all constructs were verified by DNA sequencing. Solid medium used for growing bacterial strains was nutrient agar (Oxoid), and liquid media were Luria-Bertani broth (LB) and CH medium. Chloramphenicol (5 μg/ml), erythromycin (1 μg/ml), kanamycin (5 μg/ml), spectinomycin (50 μg/ml) and tetracycline (10 μg/ml) were used for selection in *B. subtilis*, as required. Ampicillin (100 μg/ml), chloramphenicol (50 μg/ml) and kanamycin (25 μg/ml) were used for selection in *E. coli*, as required. Arabinose, IPTG and xylose were added as needed at the concentration indicated. Where indicated, 100 μM CCCP was used to collapse the proton motive force (ΔΨ and ΔpH) and 5 μM Nigericin was used to specifically dissipate ΔpH. CCCP and Nigericin were added to cells 5 min before observation and incubated with shaking at 30°C.

### Fluorescence microscopy

Cells containing fluorescent protein fusions were grown at 30°C. Xylose (0.5% w/v) was included in media to induce the expression of YFP fusions in *B. subtilis*. Cell membranes were stained by mixing 10 μl of culture with 0.2 μl of FM5-95 (200 μg/ml; Invitrogen). Nucleoids were stained with 4,6-diamidino-2-phenylindole (DAPI; Sigma), by mixing 10 μl of culture with 0.5 μl of DAPI (1 μg/ml in 50% glycerol). Cells were mounted on microscope slides covered with a thin agarose pad (1.2% w/v in dH_2_O) and were observed using a Zeiss Axiovert 200 M microscope attached to a Sony Cool-Snap HQ cooled CCD camera. Total internal reflection fluorescence (TIRF) microscopy was performed using a Nikon N-SIM microscope equipped with a Nikon APO TIRF ×100/1.49 NA objective lens. Specimens were illuminated with 488-nm solid-state lasers at 7% output, and images were acquired using an exposure time of 100 ms. Images were prepared for publication using ImageJ (http://rsb.info.nih.gov/ij).

### NBS plasmid localisation

To construct pDWA117 (8xNBS + ∽2.2 kb *tetO* array), operator arrays were obtained by digesting plasmid pLAU44 (Lau *et al*, 2003) with *Bgl*II and were ligated into *Bgl*II-digested pSG4929 (Wu *et al*, 2009). Analytical digests were used to confirm the appropriate size, number and orientation of the arrays. For plasmid localisation experiments, strains were grown in competence medium (Hamoen *et al*, 2002). Where required, xylose was included in the growth media (0.5% w/v) to induce the expression of *tetR-mCherry*. To ensure plasmid maintenance, strains containing pSG4929 or pDWA117 were propagated in the presence of erythromycin (2 μg/ml).

### Cellular fractionation

*Bacillus subtilis* cultures (50 ml) were grown in LB medium at 37°C, and at an OD_600_ of 0.4, IPTG (100 μM) was added and growth continued for 1 h. Cells were then harvested by centrifugation (5,000 *g*; 10 min; 25°C) and the pellets re-suspended in 5 ml ice-cold 100 mM Tris–HCl pH 7.5, containing a complete mini EDTA-free protease inhibitor tablet (Roche). Cells were lysed by sonication on ice and the lysate clarified by centrifugation (16,000 *g*; 10 min; 4°C). The resulting supernatant was split, half used as the total fraction and the remainder used to prepare the cytoplasmic and membrane fractions by ultra-centrifugation (195,000 *g*; 40 min; 4°C). The top 1 ml of the supernatant was taken and used as the cytoplasmic fraction. The remainder was carefully removed and the purified membrane re-suspended in an equal volume of 100 mM Tris–HCl pH 7.5. To assess the quality of the preparations, the fractions were analysed by Western blotting for the presence of known cytoplasmic (DnaA) and membrane (PBP2B) proteins.

## Supporting Information

Supplementary Movies S5

Review Process File
