# Supplementary material for: Nucleoid occlusion protein Noc recruits DNA to the bacterial cell membrane
Source: EMBO J. 2015 Jan 7;34(4):491–501. doi: 10.15252/embj.201490177 (PMC4331003; doi:10.15252/embj.201490177)
Supplement: Supplementary file 1 [file embj0034-0491-sd1.pdf]

Supplementary Information for:

Nucleoid Occlusion protein Noc recruits DNA to the Bacterial Cell Membrane

David William Adams, Ling Juan Wu & Jeff Errington

Centre for Bacterial Cell Biology, Baddiley-Clark Building, Medical School, Newcastle University, Richardson Road, Newcastle upon Tyne, NE2 4AX, United Kingdom

Supplementary Table S1 – Localisation and functionality of *noc* alleles

Protein	Localisation	Complements Δ*noc* Δ*minCD*?[a]		
		NA	0.05%	0.5%
WT	Foci over DNA	-	+	+
**N-terminus**				
K2E	DNA	-	-	+
S4A	Enhanced membrane	-	+	+
S4L	Membrane/Foci	+	+	+
F5E	DNA	-	-	-
F5A	DNA	-	-	-
R7E	DNA	-	-	+
F8A	DNA	-	-	-
F9E	DNA	-	-	-
F9A	DNA	-	-	-
K14A	WT	-	+	+
ΔN10	DNA	-	-	-
**ParB-box I**				
Q68R	DNA	-	-	-
**ParB-box II**				
G86S	DNA	-	-	-
R88A	DNA	-	-	-
R89A	DNA	-	-	-
R91A	WT - Weakened	-	+	+
**HTH DNA binding domain**				
K164A	Diffuse	-	-	-
**Combinations**				
S4L/G86S	DNA	-	-	-

[a] The ability of the indicated –YFP fusions to rescue the growth defect of a *noc minCD* mutant was assayed after growth for 18 h at 39 °C, either with no additions (NA), 0.05 % or 0.5 % w/v xylose. All proteins were produced at similar levels (Supplementary Figure S11).

Supplementary Methods

Protein expression and purification

E. coli strains BL21 (DE3), harbouring plasmids pDWA23, 31 or 32 were grown in LB at 37 °C to an OD_{600} of 0.5, at which point IPTG was added to a final concentration of 1 mM to induce protein expression. After 3 h at 30 °C, cells were harvested by centrifugation (4000 g; 10 min; 4 °C), washed once with ice-cold PBS containing 1 mM PMSF and snap-frozen in liquid nitrogen. Cell pellets were stored at -80 °C overnight to aid cell wall breakage.

Cell pellets were re-suspended in ice-cold 50 mM HEPES-KOH pH 6.8; 150 mM NaCl (Buffer A) containing a complete EDTA-free protease inhibitor tablet (Roche) and 32 mg/ml lysozyme, and incubated on ice for 1 h with gentle agitation. Cells were then lysed by sonication on ice (13 W; pulse 3) and cell-debris removed by centrifugation (31,000 g; 45 min; 4 °C). The clarified lysate was applied to a 1 ml HiTrap Q HP column (GE Healthcare) pre-equilibrated with buffer A. The column was washed with 20 column volumes (CV) of buffer A and proteins eluted using a linear gradient of 150-1000 mM NaCl over 20 CV. Fractions containing Noc were identified by absorbance at 280 nm and were analysed for purity by SDS-PAGE. Appropriate fractions were diluted in 50 mM HEPES-KOH pH 7.6; 100 mM NaCl; 1 mM EDTA (Buffer B) and applied to a 1 ml HiTrap Heparin HP Sepharose column (GE Healthcare) pre-equilibrated with the same buffer. The column was washed with 20 CV of buffer B and proteins eluted using a linear gradient of 100-1000 mM NaCl over 20 CV. Appropriate fractions were diluted in 50 mM HEPES-KOH pH 7.6; 100 mM NaCl (Buffer C) and applied to a 1 ml HiTrap Q HP column (GE Healthcare) pre-equilibrated with the same buffer. The column was washed with 20 CV of buffer C and proteins eluted using a linear gradient of 100-1000 mM NaCl over 20 CV. Fractions containing Noc were identified by absorbance at 280 nm and were analysed for purity by SDS-PAGE. Appropriate fractions were pooled (≤ 3 ml) and loaded onto HiLoad 16/60 Superdex 75 pg gel filtration column (GE Healthcare) pre-equilibrated with 30 mM HEPES-KOH pH 7.6; 300 mM NaCl (Buffer D) and eluted in the same buffer according to its hydrodynamic volume. Fractions containing

pure Noc were identified by SDS-PAGE, pooled, mixed with glycerol (10 % v/v final), aliquoted and snap-frozen in liquid nitrogen before storage at -80 °C until needed.

Analytical size-exclusion chromatography

Approximately 250 µg of purified protein was applied to a Superdex 200 10/300 GL (GE Healthcare) gel filtration column in a sample volume of 100 µl. Samples were run in 30 mM HEPES-KOH pH 7.6; 300 mM NaCl at a flow rate of 0.5 ml/min. The column was calibrated using a Gel Filtration Calibration Kit (GE Healthcare) comprising Aprotinin (6,500 Mr), Ribonuclease A (13,700 Mr), Carbonic anhydrase (29,000 Mr), Ovalbumin (43,000 Mr), Conalbumin (75,000 Mr), Aldolase (158,000 Mr) and Ferritin (440,000 Mr). The void volume was determined using Blue Dextran 2000 (> 2,000,000 Mr). The partition coefficients (K_{av}) of the protein standards were calculated according to the manufacturer's instructions and used to plot a standard curve (Supplementary Figure S3). The curve was then used to estimate the molecular weights of unknown samples by comparing the K_{av} of the sample with those of the known protein standards.

Reagents for Western blotting

Antibodies used for Western blotting were as follows: anti-FtsZ (Lucet *et al*, 2000); anti-DnaA (Scholefield *et al*, 2012); anti-PBP2B (Daniel *et al*, 2000); anti-GFP (laboratory stock, controls for specificity are shown in Supplementary Fig. S6D and E); anti-Noc (laboratory stock, controls for specificity are shown in Supplementary Figures S6E and S7C).

Plasmid construction

The sequences of all plasmids were verified by DNA sequencing (DNA Sequencing & Services, University of Dundee, Scotland). Unless otherwise specified, PCR amplification used *B. subtilis* 168 chromosomal DNA as a template and was done using Phusion® High-Fidelity DNA Polymerase (New England Biolabs). Site-directed mutagenesis was done using PfuTurbo DNA Polymerase (Agilent Technologies).

Plasmids for protein localisation in *B. subtilis*

Plasmids used for protein localisation were made using derivatives of pSG4924, which is an integrative vector (*amyE*) for creating C-terminal YFP fusions under the control of the xylose-inducible P_{xyl} promoter. All –YFP fusions used the same linker (VEVDGIDKLDIEFLQ) and Shine-Dalgarno sequence (AGGAGA). To create plasmids pDWA41 (mYFP) and pDWA42 (Noc-mYFP), the monomeric A206K mutation (Landgraf *et al*, 2012) was introduced into *yfp* by site-directed mutagenesis using pSG4924 and pSG4926, respectively, as templates. To create pDWA40, *noc* bp 1-150 encoding the first 50 amino acids of Noc was amplified by PCR. The PCR product was digested with *Bln*I and *Sal*I and cloned into pDWA41 between the *Bln*I and *Xho*I sites. To create pDWA38 (^{HCV}AH-NocNΔ10-YFP), the sequence encoding the ^{HCV}AH (MILSSLTVTQLLRRLHQWI) was included in the forward primer and was used to amplify *noc*NΔ10 by PCR. The PCR product was digested with *Bln*I and *Sal*I and cloned into pSG4924 between the *Bln*I and *Xho*I sites. The ^{HCV}AH is connected to NocNΔ10 by a GSGSGS linker sequence. To create pDWA45 (TM-NocNΔ10-mYFP), the sequence encoding the WALP23 (Nyholm *et al*, 2007) trans-membrane (TM) domain (MAWWLALALALALALALALALWWA) was included in the forward primer and was used to amplify *noc*NΔ10 by PCR. The PCR product was digested with *Bln*I and *Sal*I and cloned into pDWA41 between the *Bln*I and *Xho*I sites. The TM domain is joined to NocNΔ10 by a GSGSGS linker sequence. To create pDWA110 (NocNΔ10-mYFP), *noc*NΔ10 was amplified by PCR, digested with *Bln*I and *Sal*I and cloned

into pDWA41 between the *Bln*I and *Xho*I sites. All -YFP fusion derivatives encoding amino acid substitutions in Noc were made by site-directed mutagenesis using the templates pSG4926, pDWA42 or pDWA75, as appropriate.

Plasmids for Noc overproduction in *B. subtilis*

To construct pSG4922, *noc* was amplified by PCR, the product was digested with *Xba*I and *Cla*I and cloned into pPL82 cut with the same enzymes. pPL82 is an integrative vector (*amyE*) that carries *lacI* and the strong synthetic LacI-regulated $P_{spac(hy)}$ promoter. All constructions within pPL82 used the native *noc* Shine-Dalgarno sequence and spacer. To create pDWA79, *noc*NΔ10 was amplified by PCR, digested with *Xba*I and *Cla*I and cloned into pPL82 cut with the same enzymes. Plasmid derivatives encoding deletions or substitutions were created by site-directed mutagenesis using pSG4922 as a template.

To create pSG4943, which integrates at the *noc* locus, a 3.5 kb DNA fragment containing $P_{spac(hy)}$-*noc lacI* was isolated from pSG4922 by digestion with *Eco*RI and *Bam*HI, and then cloned into pUK19 (Wu *et al*, 2009) using the *Eco*RI and *Bam*HI sites.

Plasmids for Noc overproduction in *E. coli*

To create pDWA37, *noc* was amplified by PCR, the product digested with *Eco*RI and *Sal*I and cloned into pMG25 cut with *Eco*RI and *Sal*I. pMG25 is a pUC-derived vector that allows tightly regulated, IPTG-inducible expression. The plasmid carries *lacI*^{q} and the strong synthetic LacI-regulated $P_{A1/O3/O4}$ promoter. All pMG25 constructs used the same optimised Shine-Dalgarno sequence (AGGAGG). To create pDWA61, *noc*NΔ10 was amplified by PCR, the product digested with *Eco*RI and *Sal*I and cloned into pMG25 cut with the same enzymes. pDWA62 (NocCΔ50) was created by site-directed mutagenesis using pDWA37 as a template. To create pDWA66, *spo0J* was amplified by PCR, the

product digested with *Eco*RI and *Sal*I and cloned into pMG25 cut with the same enzymes. pDWA68 was constructed in three stages. First, *spo0J* was amplified by PCR, the product digested with *Bln*I and *Eco*RI and cloned into pSG1728 digested with the same enzymes. Second, the sequence encoding the N-terminal 30 amino acids of Noc was cloned into this plasmid between *Bln*I (within vector) and *Stu*I (within *spo0J*). Third, using this intermediate plasmid as a template, *noc*$^{bp1\text{-}90}$-*spo0J* was amplified by PCR, the product digested with *Eco*RI and *Sal*I and cloned into pMG25 cut with the same enzymes. Note that in order to avoid any potential complications due to Spo0J or Noc30-Spo0J proteins binding to the *parS* site within the *spo0J* gene, plasmids pDWA66 and pDWA68 were assembled using a *parS*-minus allele of *spo0J*, which contains changes in 7 bp (of 16) (WT, TGTTCCACGTGAAACA; *parS*-minus, CGTGCCCAGGGAGACC) in the *parS* site within *spo0J* without affecting the amino acid sequence of the gene product (Lin & Grossman, 1998). To create pDWA69, ^{HCV}AH-*noc*NΔ10 was amplified by PCR using pDWA38 as a template. The PCR product was digested with *Eco*RI and *Sal*I and cloned into pMG25 cut with the same enzymes.

GFP-JunZ-MTS derivatives

pDWA20 encodes an arabinose inducible fusion protein comprised of GFP, JunLZ and the first 30 amino acids of Noc, with a triglycine linker between JunLZ and Noc$^{1\text{-}30}$. JunLZ is a homodimerization-enhanced mutant of the leucine zipper motif from the c-Jun transcription factor (Szeto *et al*, 2003). To create pDWA20 the sequence encoding the *E. coli* MinD membrane targeting sequence (MTS) in pTS37 was replaced with the sequence encoding the first 30 amino acids of Noc by PCR.

Plasmids for protein purification

To create pDWA23, *noc* was amplified by PCR and cut with *Bsp*HI and *Xho*I. The digested PCR product was cloned between the *Nco*I and *Xho*I sites of pET16b such that the native protein (*i.e.*

without any tag) is produced under the control of the T7 promoter. To create pDWA31, *noc*NΔ10 was amplified by PCR, digested with *Nco*I and *Xho*I and cloned into pET16b cut with the same enzymes. pDWA32 (NocCΔ50) was created by site-directed mutagenesis of pDWA23.

Supplementary Table S2 - Strains and plasmids

Strain/Plasmid	Relevant genotype[a]	Reference / Origin[b]
B. subtilis		
168CA	*trpC2*	Laboratory stock
HS13	Δ*atpB*::*erm*	(Strahl & Hamoen, 2010)
3309	*trpC2* Δ*minCD*::*kan*	(Wu & Errington, 2004)
4171	*trpC2* Δ*noc*::*tet*, Ω*amyE*::(*spc* P_{xyl}-*noc*NΔ10-*yfp*)	J Schneeweiss, unpublished
4702	*trpC2* Δ*noc*::*tet*, Ω*amyE*::(*spc* P_{xyl}-*noc*-*yfp*)	(Wu *et al*, 2009)
4712	*trpC2* Ω *noc*::pSG4934 (*kan* P_{spac}-*noc'*)	(Wu *et al*, 2009)
4705	*trpC2* Δ*noc*::*tet*, Ω*amyE*::(*spc* P_{xyl}-*noc*-*yfp*), Ω*cgeD*::pAT12(*cat lacO*x256) Ω*thrC*::P_{pen}-*lacIA* 11-*cfp*(W7) *mls*	(Wu *et al*, 2009)
HM773	*trpC2* Ω*amyE*::(*spc* P_{xyl}-*tetR-mCherry*)	H Murray, unpublished
DWA66	*trpC2* Ω *noc*::pSG4934 (*kan* P_{spac}-*noc'*)	4712 > 168 (Km)
DWA78	*trpC2* Ω *noc*::pSG4934 (*kan* P_{spac}-*noc'*) + pSG4929	pSG4929 > DWA66 (Em)
DWA103	*trpC2* Δ*noc*::*tet*, Ω*amyE*::(*spc* P_{xyl}-*noc*-*yfp*), Δ*atpB*::*erm*	HS13 >4702 (Em)
DWA117	*trpC2* Δ*noc*::*tet*	4702 > 168 (Te)
DWA119	*trpC2* Δ*noc*::*tet*, Ω*amyE*::(*cat lacI* $P_{spac(hy)}$-*noc*)	pSG4922 > DWA117 (Cm)
DWA127	*trpC2* Ω*noc*:($P_{spac(hy)}$-*noc lacI kan*)	pSG4943 > 168 (Km)
DWA146	*trpC2* Δ*noc*::*tet*, Ω*amyE*::(*spc* P_{xyl}-*noc*NΔ10-*yfp*)	4171 > DWA117 (Sp)
DWA193	*trpC2* Δ*noc*::*tet*, Ω*amyE*::(*spc* P_{xyl}-^{HCV}AH-*noc*NΔ10-*yfp*)	pDWA38 > DWA117 (Sp)
DWA195	*trpC2* Δ*noc*::*tet*, Ω*amyE*::(*spc* P_{xyl}-^{HCV}AH-*gfp*)	H Strahl, unpublished
DWA206	*trpC2* Δ*noc*::*tet*, Ω*amyE*::(*spc* P_{xyl}-*noc*-m*yfp*)	pDWA42 > DWA117 (Sp)
DWA211	*trpC2* Δ*noc*::*tet*, Ω*amyE*::(*spc* P_{xyl}-*noc*F5E-*yfp*)	pDWA43 > DWA117 (Sp)
DWA212	*trpC2* Δ*noc*::*tet*, Ω*amyE*::(*spc* P_{xyl}-*noc*R7E-*yfp*)	pDWA44 > DWA117 (Sp)
DWA215	*trpC2* Δ*noc*::*tet*, Ω*amyE*::(*spc* P_{xyl}-*noc*50-m*yfp*)	pDWA40 > DWA117 (Sp)
DWA225	*trpC2* Ω*amyE*::(*spc* P_{xyl}-*noc*50-m*yfp*)	DWA215 > 168 (Sp)
DWA226	*trpC2* Δ*noc*::*tet*, Ω*amyE*::(*cat lacI* $P_{spac(hy)}$-*noc*F5E)	pDWA46 > DWA117 (Cm)
DWA227	*trpC2* Δ*noc*::*tet*, Ω*amyE*::(*cat lacI* $P_{spac(hy)}$-*noc*R7E)	pDWA47 > DWA117 (Cm)
DWA282	*trpC2* Δ*noc*::*tet*, Ω*amyE*::(*cat lacI* $P_{spac(hy)}$-*noc*NΔ10)	pDWA79 > DWA117 (Cm)
DWA283	*trpC2* Δ*noc*::*tet*, Ω*amyE*::(*cat lacI* $P_{spac(hy)}$-*noc*Q68R)	pDWA78 > DWA117 (Cm)
DWA284	*trpC2* Δ*noc*::*tet*, Ω*amyE*::(*cat lacI* $P_{spac(hy)}$-*noc*G86S)	pDWA82 > DWA117 (Cm)
DWA285	*trpC2* Δ*noc*::*tet*, Ω*amyE*::(*spc* P_{xyl}-*noc*Q68R-m*yfp*)	pDWA81 > DWA117 (Sp)
DWA286	*trpC2* Δ*noc*::*tet*, Ω*amyE*::(*spc* P_{xyl}-*noc*G86S-m*yfp*)	pDWA75 > DWA117 (Sp)
DWA302	*trpC2* Δ*noc*::*tet*, Ω*amyE*::(*spc* P_{xyl}-^{HCV}AH-*noc*NΔ10)	pDWA74 > DWA117 (Sp)
DWA306	*trpC2* Δ*minCD*::*kan*	3309 > 168 (Km)
DWA307	*trpC2* Δ*noc*::*tet*, Δ*minCD*::*kan*, Ω*amyE*::(*spc* P_{xyl}-^{HCV}AH-*noc*NΔ10)	DWA306 > DWA302 (Km)
DWA316	*trpC2* Δ*noc*::*tet*, Ω*amyE*::(*spc* P_{xyl}-*noc*K2E-m*yfp*)	pDWA91 > DWA117 (Sp)
DWA318	*trpC2* Δ*noc*::*tet*, Ω*amyE*::(*spc* P_{xyl}-*noc*F9E-m*yfp*)	pDWA95 > DWA117 (Sp)
DWA322	*trpC2* Δ*noc*::*tet*, Ω*amyE*::(*spc* P_{xyl}-*noc*F5A-m*yfp*)	pDWA96 > DWA117 (Sp)
DWA323	*trpC2* Δ*noc*::*tet*, Ω*amyE*::(*spc* P_{xyl}-*noc*F8A-m*yfp*)	pDWA97 > DWA117 (Sp)
DWA325	*trpC2* Δ*noc*::*tet*, Ω*amyE*::(*spc* P_{xyl}-*noc*F9A-m*yfp*)	pDWA98 > DWA117 (Sp)
DWA328	*trpC2* Δ*noc*::*tet*, Ω*amyE*::(*spc* P_{xyl}-*noc*S4A-m*yfp*)	pDWA101 > DWA117 (Sp)
DWA329	*trpC2* Δ*noc*::*tet*, Ω*amyE*::(*spc* P_{xyl}-*noc*S4L-m*yfp*)	pDWA102 > DWA117 (Sp)
DWA343	*trpC2* Δ*noc*::*tet*, Ω*amyE*::(*cat lacI* $P_{spac(hy)}$-*noc*ΔK2)	pSG4939 > DWA117 (Cm)
DWA344	*trpC2* Δ*noc*::*tet*, Ω*amyE*::(*cat lacI* $P_{spac(hy)}$-*noc*K2A)	pSG4940 > DWA117 (Cm)

DWA346	*trpC2* Δ*noc*::*tet*, Ω*amyE*::(*cat lacI* P$_{spac(hy)}$-*noc*ΔF5,S6)	pSG4941 > DWA117 (Cm)
DWA347	*trpC2* Δ*noc*::*tet*, Ω*amyE*::(*cat lacI* P$_{spac(hy)}$-*noc*R7A)	pSG4937 > DWA117 (Cm)
DWA348	*trpC2* Δ*noc*::*tet*, Ω*amyE*::(*cat lacI* P$_{spac(hy)}$-*noc*K14A)	pSG4938 > DWA117 (Cm)
DWA349	*trpC2* Δ*noc*::*tet*, Ω*amyE*::(*spc* P$_{xyl}$-*noc*S4L,G86S-m*yfp*)	pDWA103 > DWA117 (Sp)
DWA350	*trpC2* Δ*noc*::*tet*, Δ*minCD*::*kan*	DWA306 > DWA117 (Km)
DWA351	*trpC2* Δ*noc*::*tet*, Δ*minCD*::*kan*, Ω*amyE*::(*cat lacI* P$_{spac(hy)}$-*noc*)	DWA306 > DWA119 (Km)
DWA352	*trpC2* Δ*noc*::*tet*, Δ*minCD*::*kan*, Ω*amyE*::(*cat lacI* P$_{spac(hy)}$-*noc*NΔ10)	DWA306 > DWA282 (Km)
DWA357	*trpC2* Δ*noc*::*tet*, Δ*minCD*::*kan*, Ω*amyE*::(*cat lacI* P$_{spac(hy)}$-*noc*Q68R)	DWA306 > DWA283 (Km)
DWA358	*trpC2* Δ*noc*::*tet*, Δ*minCD*::*kan*, Ω*amyE*::(*cat lacI* P$_{spac(hy)}$-*noc*G86S)	DWA306 > DWA284 (Km)
DWA362	*trpC2* Δ*minCD*::*kan*, Ω*amyE*::(*cat lacI* P$_{spac(hy)}$-*noc*)	DWA119 > DWA306 (Cm)
DWA363	*trpC2* Δ*minCD*::*kan*, Ω*amyE*::(*cat lacI* P$_{spac(hy)}$-*noc*NΔ10)	DWA282 > DWA306 (Cm)
DWA364	*trpC2* Δ*minCD*::*kan*, Ω*amyE*::(*cat lacI* P$_{spac(hy)}$-*noc*Q68R)	DWA283 > DWA306 (Cm)
DWA365	*trpC2* Δ*minCD*::*kan*, Ω*amyE*::(*cat lacI* P$_{spac(hy)}$-*noc*G86S)	DWA284 > DWA306 (Cm)
DWA370	*trpC2* Ω*noc*:(P$_{spac(hy)}$-*noc lacI kan*), Ω*amyE*::(*spc* P$_{xyl}$-*noc*NΔ10-*yfp*)	DWA146 > DWA127 (Sp)
DWA371	*trpC2* Ω*noc*:(P$_{spac(hy)}$-*noc lacI kan*), Ω*amyE*::(*spc* P$_{xyl}$-*noc*G86S-m*yfp*)	DWA286 > DWA127 (Sp)
DWA382	*trpC2* Δ*noc*::*tet*, Ω*amyE*::(*spc* P$_{xyl}$-*noc*NΔ10-m*yfp*)	pDWA110 > DWA117 (Sp)
DWA397	*trpC2* Δ*noc*::*tet*, Ω*amyE*::(*spc* P$_{xyl}$-*noc*-m*yfp*), Ω*cgeD*::pAT12(*cat lacO*x256) Ω*thrC*::P$_{pen}$-*lacIA* 11-*cfp*(W7) *mls*	4705 > DWA206 (Em), then 4705 > resulting strain (Cm)
DWA398	*trpC2* Δ*noc*::*tet*, Ω*amyE*::(*spc* P$_{xyl}$-*noc*NΔ10-m*yfp*), Ω*cgeD*::pAT12(*cat lacO*x256) Ω*thrC*::P$_{pen}$-*lacIA* 11-*cfp*(W7) *mls*	4705 > DWA382 (Em), then 4705 > resulting strain (Cm)
DWA399	*trpC2* Δ*noc*::*tet*, Ω*amyE*::(*spc* P$_{xyl}$-*noc*Q68R-m*yfp*), Ω*cgeD*::pAT12(*cat lacO*x256) Ω*thrC*::P$_{pen}$-*lacIA* 11-*cfp*(W7) *mls*	4705 > DWA285 (Em), then 4705 > resulting strain (Cm)
DWA400	*trpC2* Δ*noc*::*tet*, Ω*amyE*::(*spc* P$_{xyl}$-*noc*G86S-m*yfp*), Ω*cgeD*::pAT12(*cat lacO*x256) Ω*thrC*::P$_{pen}$-*lacIA* 11-*cfp*(W7) *mls*	4705 > DWA286 (Em), then 4705 > resulting strain (Cm)
DWA427	*trpC2* Ω*noc*::pSG4934 (*kan* P$_{spac}$-*noc'*) Ω*amyE*::(*spc* P$_{xyl}$-*tetR-mCherry*)	4712 > HM773 (Km)
DWA429	*trpC2* Ω*noc*::pSG4934 (*kan* P$_{spac}$-*noc'*) Ω*amyE*::(*spc* P$_{xyl}$-*tetR-mCherry*) + pDWA117	pDWA117 > DWA427 (Em)
DWA519	*trpC2* Δ*noc::tet,* Ω*amyE*::(*spc* P$_{xyl}$-*noc-myfp*) + pSG4929	pSG4929 > DWA206 (Em)
DWA520	*trpC2* Δ*noc::tet,* Ω*amyE*::(*spc* P$_{xyl}$-*noc*Q68R-*myfp*) + pSG4929	pSG4929 > DWA382 (Em)
DWA521	*trpC2* Δ*noc::tet,* Ω*amyE*::(*spc* P$_{xyl}$-*noc*G86S-*myfp*) + pSG4929	pSG4929 > DWA285 (Em)
DWA522	*trpC2* Δ*noc::tet,* Ω*amyE*::(*spc* P$_{xyl}$-*noc*NΔ10-*myfp*) + pSG4929	pSG4929 > DWA286 (Em)
DWA523	*trpC2* Δ*noc*::*tet*, Ω*amyE*::(*cat lacI* P$_{spac(hy)}$-*noc*) + pSG4929	pSG4929 > DWA119 (Em)

DWA524	*trpC2* Δ*noc*::*tet*, Ω*amyE*::(*cat lacI* $P_{spac(hy)}$-*noc*NΔ10) + pSG4929	pSG4929 > DWA282 (Em)
DWA525	*trpC2* Δ*noc*::*tet*, Ω*amyE*::(*cat lacI* $P_{spac(hy)}$-*noc*Q68R) + pSG4929	pSG4929 > DWA283 (Em)
DWA526	*trpC2* Δ*noc*::*tet*, Ω*amyE*::(*cat lacI* $P_{spac(hy)}$-*noc*G86S) + pSG4929	pSG4929 > DWA284 (Em)
DWA545	*trpC2* Δ*noc::tet,* Ω*amyE*::(*spc* P_{xyl}-*noc*R88A-*myfp*)	pDWA140 > DWA117 (Sp)
DWA546	*trpC2* Δ*noc::tet,* Ω*amyE*::(*spc* P_{xyl}-*noc*R89A-*myfp*)	pDWA141 > DWA117 (Sp)
DWA547	*trpC2* Δ*noc::tet,* Ω*amyE*::(*spc* P_{xyl}-*noc*R91A-*myfp*)	pDWA142 > DWA117 (Sp)
DWA548	*trpC2* Δ*noc::tet,* Ω*amyE*::(*spc* P_{xyl}-TM-NΔ10-*myfp*)	pDWA45 > DWA117 (Sp)
DWA551	*trpC2* Δ*noc::tet,* Ω*amyE*::(*cat* $P_{spac(hy)}$-*noc* R88A)	pDWA144 > DWA117 (Cm)
DWA552	*trpC2* Δ*noc::tet,* Ω*amyE*::(*cat* $P_{spac(hy)}$-*noc* R89A)	pDWA145 > DWA117 (Cm)
DWA553	*trpC2* Δ*noc::tet,* Ω*amyE*::(*cat* $P_{spac(hy)}$-*noc* R91A)	pDWA146 > DWA117 (Cm)
DWA559	*trpC2* Ω*amyE*::(*cat* $P_{spac(hy)}$-*noc* R88A), Δ*minCD::kan*	DWA551 > DWA306 (Cm)
DWA560	*trpC2* Ω*amyE*::(*cat* $P_{spac(hy)}$-*noc* R89A), Δ*minCD::kan*	DWA552 > DWA306 (Cm)
DWA561	*trpC2* Ω*amyE*::(*cat* $P_{spac(hy)}$-*noc* R91A), Δ*minCD::kan*	DWA553 > DWA306 (Cm)
DWA562	*trpC2* Δ*noc::tet,* Ω*amyE*::(*spc* P_{xyl}-TM-NΔ10-*myfp*), Δ*minCD::kan*	DWA350 > DWA548 (Km)
DWA564	*trpC2* Δ*noc::tet,* Ω*amyE*::(*spc* P_{xyl}-*noc-myfp*), Δ*minCD::kan*	DWA350 > DWA206 (Km)
DWA566	*trpC2* Δ*noc*::*tet*, Ω*amyE*::(*spc* P_{xyl}-*noc*NΔ10-m*yfp*), Δ*minCD::kan*	DWA350 > DWA382 (Km)
DWA568	*trpC2* Δ*noc*::*tet*, Ω*amyE*::(*spc* P_{xyl}-*noc*G86S-m*yfp*), Δ*minCD::kan*	DWA350 > DWA286 (Km)
DWA570	*trpC2* Δ*noc*::*tet*, Ω*amyE*::(*spc* P_{xyl}-*noc*K2E-m*yfp*), Δ*minCD::kan*	DWA350 > DWA316 (Km)
DWA574	*trpC2* Δ*noc*::*tet*, Ω*amyE*::(*spc* P_{xyl}-*noc*S4A-m*yfp*), Δ*minCD::kan*	DWA350 > DWA328 (Km)
DWA576	*trpC2* Δ*noc*::*tet*, Ω*amyE*::(*spc* P_{xyl}-*noc*S4L-m*yfp*), Δ*minCD::kan*	DWA350 > DWA329 (Km)
DWA578	*trpC2* Δ*noc*::*tet*, Ω*amyE*::(*spc* P_{xyl}-*noc*F5E-*yfp*), Δ*minCD::kan*	DWA350 > DWA211 (Km)
DWA580	*trpC2* Δ*noc*::*tet*, Ω*amyE*::(*spc* P_{xyl}-*noc*R7E-*yfp*), Δ*minCD::kan*	DWA350 > DWA212 (Km)
DWA582	*trpC2* Δ*noc*::*tet*, Ω*amyE*::(*spc* P_{xyl}-*noc*F9E-m*yfp*), Δ*minCD::kan*	DWA350 > DWA318 (Km)
DWA584	*trpC2* Δ*noc*::*tet*, Ω*amyE*::(*spc* P_{xyl}-*noc*F9A-m*yfp*), Δ*minCD::kan*	DWA350 > DWA325 (Km)
DWA588	*trpC2* Δ*noc*::*tet*, Ω*amyE*::(*spc* P_{xyl}-*noc*S4L,G86S-m*yfp*), Δ*minCD::kan*	DWA350 > DWA349 (Km)
DWA590	*trpC2* Δ*noc*::*tet*, Ω*amyE*::(*spc* P_{xyl}-*noc*Q68R-m*yfp*), Δ*minCD::kan*	DWA350 > DWA285 (Km)
DWA598	*trpC2* Δ*noc::tet,* Ω*amyE*::(*spc* P_{xyl}-*noc*R88A-*myfp*), Δ*minCD::kan*	DWA350 > DWA545 (Km)
DWA600	*trpC2* Δ*noc::tet,* Ω*amyE*::(*spc* P_{xyl}-*noc*R89A-*myfp*), Δ*minCD::kan*	DWA350 > DWA546 (Km)
DWA602	*trpC2* Δ*noc::tet,* Ω*amyE*::(*spc* P_{xyl}-*noc*R91A-*myfp*), Δ*minCD::kan*	DWA350 > DWA547 (Km)
DWA606	*trpC2* Δ*noc::tet,* Ω*amyE*::(*spc* P_{xyl}-*noc*K164A-*myfp*)	pDWA147 > DWA117 (Sp)
DWA611	*trpC2* Δ*noc::tet,* Ω*amyE*::(*spc* P_{xyl}-*noc*F5A-m*yfp*), Δ*minCD::kan*	DWA350 > DWA322 (Km)

DWA613	*trpC2* Δ*noc*::*tet*, Ω*amyE*::(*spc* P_{xyl}-*noc*F8A-m*yfp*), Δ*minCD::kan*	DWA350 > DWA323 (Km)
DWA615	*trpC2* Δ*noc::tet,* Ω*amyE*::(*spc* P_{xyl}-*noc*K164A-*myfp*)	DWA350 > DWA606 (Km)
DWA623	*trpC2* Δ*noc::tet,* Ω*amyE*::(*cat* $P_{spac(hy)}$-*noc* R88A) + pSG4929	pSG4929 > DWA551 (Em)
DWA624	*trpC2* Δ*noc::tet,* Ω*amyE*::(*cat* $P_{spac(hy)}$-*noc* R89A) + pSG4929	pSG4929 > DWA552 (Em)
DWA625	*trpC2* Δ*noc::tet,* Ω*amyE*::(*cat* $P_{spac(hy)}$-*noc* R91A) + pSG4929	pSG4929 > DWA553 (Em)
DWA626	*trpC2* Δ*noc::tet,* Ω*amyE*::(*spc* P_{xyl}-*noc*R88A-*myfp*) + pSG4929	pSG4929 > DWA545 (Em)
DWA627	*trpC2* Δ*noc::tet,* Ω*amyE*::(*spc* P_{xyl}-*noc*R89A-*myfp*) + pSG4929	pSG4929 > DWA546 (Em)
DWA628	*trpC2* Δ*noc::tet,* Ω*amyE*::(*spc* P_{xyl}-*noc*R91A-*myfp*) + pSG4929	pSG4929 > DWA547 (Em)
DWA629	*trpC2* Δ*noc*::*tet*, Ω*amyE*::(*spc* P_{xyl}-^{HCV}AH-*gfp*), Δ*minCD::kan*	DWA195 > DWA117 (Sp) then DWA350 > resulting strain (Km)
DWA634	*trpC2* Δ*noc*::*tet*, Ω*amyE*::(*spc* P_{xyl}-*noc*K14A-*yfp*)	pSG4944 > DWA117 (Sp)
DWA635	*trpC2* Δ*noc*::*tet*, Ω*amyE*::(*spc* P_{xyl}-*noc*K14A-*yfp*), Δ*minCD::kan*	DWA350 > DWA634 (Km)
DWA636	*trpC2* Δ*noc*::*tet*, Ω*amyE*::(*spc* P_{xyl}-^{HCV}AH-*noc*NΔ10-*yfp*), Δ*minCD::kan*	DWA350 > DWA193 (Km)
E. coli		
MC1000	Δ(*ara-leu*) Δ*lac rps*L150	Laboratory stock
MC1000Δ*minCD*	MC1000 Δ*minCD*::*kan*	(Ebersbach *et al*, 2008)
MC1000Δ*slmA*	MC1000 Δ*slmA*::*cat*	(Ebersbach *et al*, 2008)
MC1000Δ*sulA*	MC1000 Δ*sulA*	(Ebersbach *et al*, 2008)
MG1655	Wild-type *E. coli* K-12	Laboratory stock
DWA260	MG1655: pMG25	pMG25 > MG1655 (Amp)
DWA261	MG1655: pDWA37	pDWA37 > MG1655 (Amp)
DWA266	MG1655: pDWA61	pDWA61 > MG1655 (Amp)
DWA267	MG1655: pDWA62	pDWA62 > MG1655 (Amp)
DWA270	MG1655: pDWA69	pDWA69 > MG1655 (Amp)
DWA271	MG1655: pDWA66	pDWA66 > MG1655 (Amp)
DWA272	MG1655: pDWA68	pDWA68 > MG1655 (Amp)
DWA334	MC1000, pDWA37	pDWA37 > MC1000 (Amp)
DWA335	MC1000 Δ*slmA*::*cat*, pDWA37	pDWA37 > MC1000Δ*minCD* (Amp)
DWA336	MC1000 Δ*minCD*::*kan*, pDWA37	pDWA37 > MC1000Δ*slmA* (Amp)
DWA337	MC1000 Δ*sulA*, pDWA37	pDWA37 > MC1000Δ*sulA* (Amp)

Plasmids		
pET16b	*bla lacI* P_{T7}-10xhis	Novagen
pSG4924	*bla amyE' spc* P_{xyl}-*yfp*^{mut1} *'amyE*	(Wu *et al*, 2009)
pSG4926	*bla amyE' spc* P_{xyl}-*noc-yfp*^{mut1} *'amyE*	(Wu *et al*, 2009)
pSG4929	P_{spaS} 8xNBS(*ydbO*) *erm*	(Wu *et al*, 2009)
pPL82	*bla amyE' cat lacI* $P_{spac(hy)}$- *'amyE*	(Quisel *et al*, 2001)
pMG25	*bla lacI*^{q} $P_{A1/O4/O3}$	(Christensen-Dalsgaard *et al*, 2008)
pTS37	*cat* P_{ara}-*gfp-junLZ-Ec*MinD MTS$^{256–270}$	(Szeto *et al*, 2003)
pLAU44	*tetO*; *Gm*^{R}	(Lau *et al*, 2003)
pDWA20	*cat* P_{ara}-*gfp-junLZ-Bs*Noc MTS$^{1\text{-}30}$	This work
pDWA23	*bla lacI* P_{T7}-*noc*	This work
pDWA31	*bla lacI* P_{T7}-*noc* NΔ10	This work
pDWA32	*bla lacI* P_{T7}-*noc* CΔ50	This work
pDWA37	*bla lacI*^{q} $P_{A1/O4/O3}$-*noc*	This work
pDWA38	*bla amyE' spc* P_{xyl}-^{HCV}AH-*noc*NΔ10-*yfp 'amyE*	This work
pDWA40	*bla amyE' spc* P_{xyl}-noc^{AA}1-50-m*yfp 'amyE*	This work
pDWA41	*bla amyE' spc* P_{xyl}-m*yfp 'amyE*	This work
pDWA42	*bla amyE' spc* P_{xyl}-*noc*-m*yfp 'amyE*	This work
pDWA43	*bla amyE' spc* P_{xyl}-*noc*F5E-*yfp*^{mut1} *'amyE*	This work
pDWA44	*bla amyE' spc* P_{xyl}-*noc*R7E-*yfp*^{mut1} *'amyE*	This work
pDWA45	*bla amyE' spc* P_{xyl}-TM-*noc*NΔ10-m*yfp 'amyE*	This work
pDWA46	*bla amyE' cat lacI* $P_{spac(hy)}$-*noc*F5E *'amyE*	This work
pDWA47	*bla amyE' cat lacI* $P_{spac(hy)}$-*noc*R7E *'amyE*	This work
pDWA61	*bla lacI*^{q} $P_{A1/O4/O3}$-*noc* NΔ10	This work
pDWA62	*bla lacI*^{q} $P_{A1/O4/O3}$-*noc* CΔ50	This work
pDWA66	*bla lacI*^{q} $P_{A1/O4/O3}$-*spo0J*(*parS*-)	This work
pDWA68	*bla lacI*^{q} $P_{A1/O4/O3}$-*noc*30-*spo0J*(*parS*-)	This work
pDWA69	*bla lacI*^{q} $P_{A1/O4/O3}$-^{HCV}AH-*noc*NΔ10	This work
pDWA74	*bla amyE' spc* P_{xyl}-^{HCV}AH-*noc*NΔ10 *'amyE*	This work
pDWA75	*bla amyE' spc* P_{xyl}-*noc*G86S-m*yfp 'amyE*	This work
pDWA78	*bla amyE' cat lacI* $P_{spac(hy)}$-*noc*Q68R *'amyE*	This work
pDWA79	*bla amyE' cat lacI* $P_{spac(hy)}$-*noc*NΔ10 *'amyE*	This work
pDWA81	*bla amyE' spc* P_{xyl}-*noc*Q68R-m*yfp 'amyE*	This work
pDWA82	*bla amyE' cat lacI* $P_{spac(hy)}$-*noc*G86S *'amyE*	This work
pDWA91	*bla amyE' spc* P_{xyl}-*noc*K2E-m*yfp 'amyE*	This work
pDWA95	*bla amyE' spc* P_{xyl}-*noc*F9E-m*yfp 'amyE*	This work
pDWA96	*bla amyE' spc* P_{xyl}-*noc*F5A-m*yfp 'amyE*	This work
pDWA97	*bla amyE' spc* P_{xyl}-*noc*F8A-m*yfp 'amyE*	This work
pDWA98	*bla amyE' spc* P_{xyl}-*noc*F9A-m*yfp 'amyE*	This work
pDWA101	*bla amyE' spc* P_{xyl}-*noc*S4A-m*yfp 'amyE*	This work
pDWA102	*bla amyE' spc* P_{xyl}-*noc*S4L-m*yfp 'amyE*	This work
pDWA103	*bla amyE' spc* P_{xyl}-*noc*S4L,G86S-m*yfp 'amyE*	This work
pDWA110	*bla amyE' spc* P_{xyl}-*noc*NΔ10-m*yfp 'amyE*	This work
pDWA117	P_{spaS} 8xNBS(*ydbO*) *erm* + *tetO* array	This work
pDWA140	*bla amyE' spc* P_{xyl}-*noc*R88A-m*yfp 'amyE*	This work
pDWA141	*bla amyE' spc* P_{xyl}-*noc*R89A-m*yfp 'amyE*	This work
pDWA142	*bla amyE' spc* P_{xyl}-*noc*R91A-m*yfp 'amyE*	This work
pDWA144	*bla amyE' cat lacI* $P_{spac(hy)}$-*noc*R88A *'amyE*	This work
pDWA145	*bla amyE' cat lacI* $P_{spac(hy)}$-*noc*R89A *'amyE*	This work
pDWA146	*bla amyE' cat lacI* $P_{spac(hy)}$-*noc*R91A *'amyE*	This work

pDWA147	*bla amyE' spc* P_{xyl}-*noc*K164A-m*yfp 'amyE*	This work
pSG4922	*bla amyE' cat lacI* $P_{spac(hy)}$-*noc 'amyE*	This work
pSG4937	*bla amyE' cat lacI* $P_{spac(hy)}$-*noc*R7A *'amyE*	This work
pSG4938	*bla amyE' cat lacI* $P_{spac(hy)}$-*noc*K14A *'amyE*	This work
pSG4939	*bla amyE' cat lacI* $P_{spac(hy)}$-*noc*ΔK2 *'amyE*	This work
pSG4940	*bla amyE' cat lacI* $P_{spac(hy)}$-*noc*K2A *'amyE*	This work
pSG4941	*bla amyE' cat lacI* $P_{spac(hy)}$-*noc*ΔF5,S6 *'amyE*	This work
pSG4943	*bla noc*::($P_{spac(hy)-}$*noc lacI kan*)	This work
pSG4944	*bla amyE' spc* P_{xyl}-*noc*K14A-*yfp 'amyE*	This work

[a]Resistance gene abbreviations: *bla*, ampicillin; *cat*, chloramphenicol; *erm*, erythromycin; *kan*, kanamycin; *spc*, spectinomycin; *tet*, tetracycline.

[b]For strains constructed by transformation, the source of the DNA used in the transformation is given first. The recipient strain is indicated after the arrow, with the selected marker in parentheses: Amp, ampicillin; Cm, chloramphenicol; Em, erythromycin; Km, kanamycin; Sp, spectinomycin; Te, tetracycline.

Supplementary Figures

Supplementary Figure S1

(**A**-**D**) CCCP-induced Noc delocalisation is due to the loss of ΔΨ. Cells of strain 4702 expressing Noc-YFP were examined either without additions (**A**) or 5 min after treatment with Nigericin (5 µM) (**B**). (**C**-**D**) Effect of CCCP on Noc-YFP localisation in a F_1F_0 ATP synthase-deficient background. To rule out the possibility that the altered Noc localisation resulted from the drop in cellular ATP levels that occurs upon CCCP treatment (Strahl & Hamoen, 2010) due to the ability of the F_1F_o ATP-synthase to function as an ATP-driven proton pump (Hicks *et al*, 1994), the experiment was repeated in an F_1F_o ATP-synthase deficient strain that is able to maintain normal ATP levels when grown in rich medium (Santana *et al*, 1994; Strahl & Hamoen, 2010). Cells of strain DWA103 (*atpB*::*erm*) expressing Noc-YFP were grown in LB + 0.4 % (w/v) glucose and examined either after no additions (**C**) or 5 min after treatment with CCCP (100 µM) (**D**). The corresponding phase contrast images are shown below each panel. Scale bar = 5 µm.

Figure S1

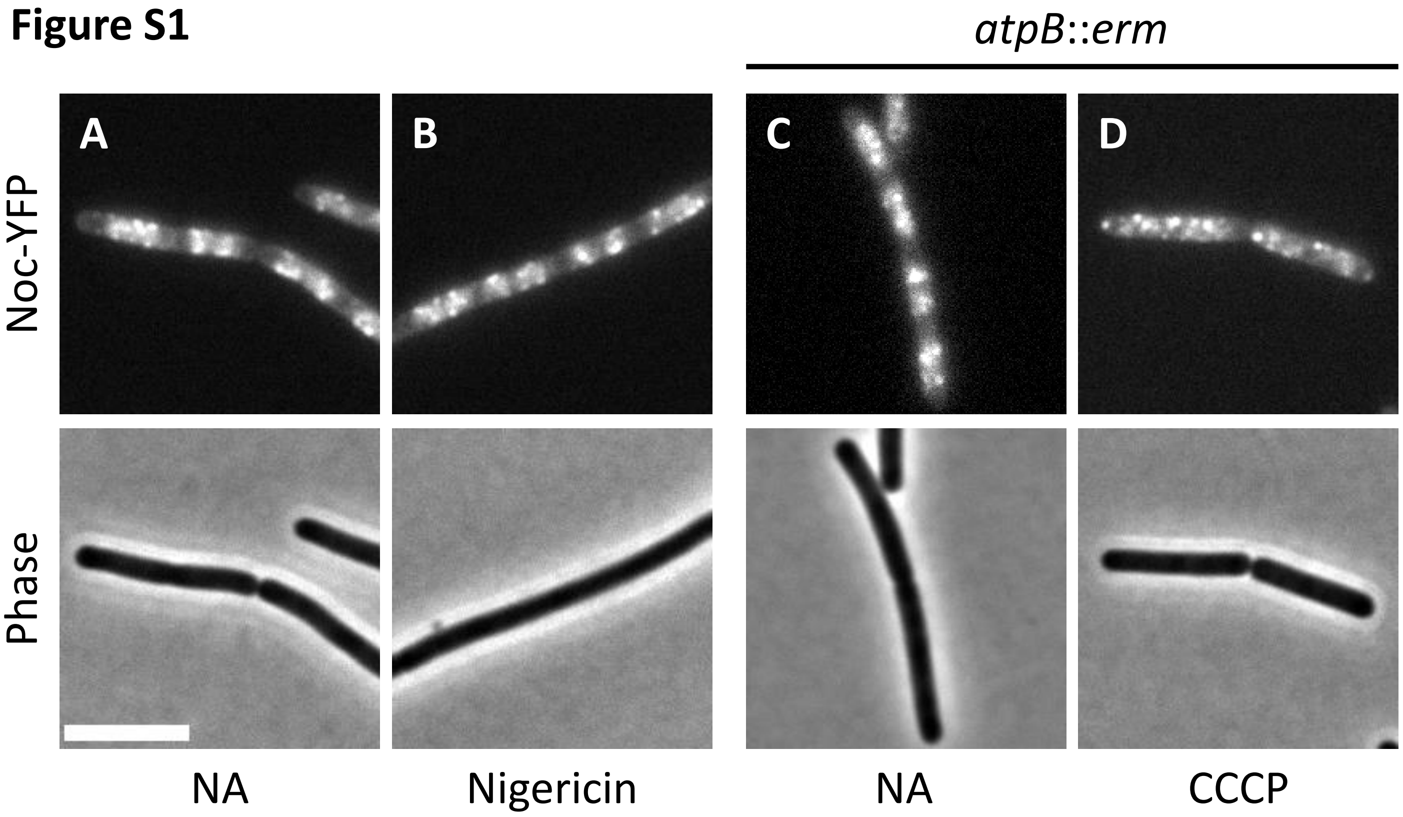

Supplementary Figure S2

(**A**) Multiple sequence alignment of Noc homologues. The *B. subtilis* Noc N-terminus was used as a query sequence for BLAST-P against a non-redundant protein database. The top 100 sequences returned were aligned using Clustal Omega. The figure was prepared using Jalview and is coloured according to percentage identity, as depicted in the key. The N-terminus is highlighted in red.

(**B**) Multiple sequence alignment of Noc and Spo0J homologues. Reference sequences were aligned using Clustal Omega. The figure was prepared using Jalview and is coloured according to percentage identity, as depicted in the key. Species abbreviations: *B. subtilis*, ***Bs***; *B. anthracis*, ***Ba***; *B. cereus*, ***Bc***; *B. amyloliquefaciens*, ***Bam***; *B. licheniformis*, ***Bl***.

Figure S2

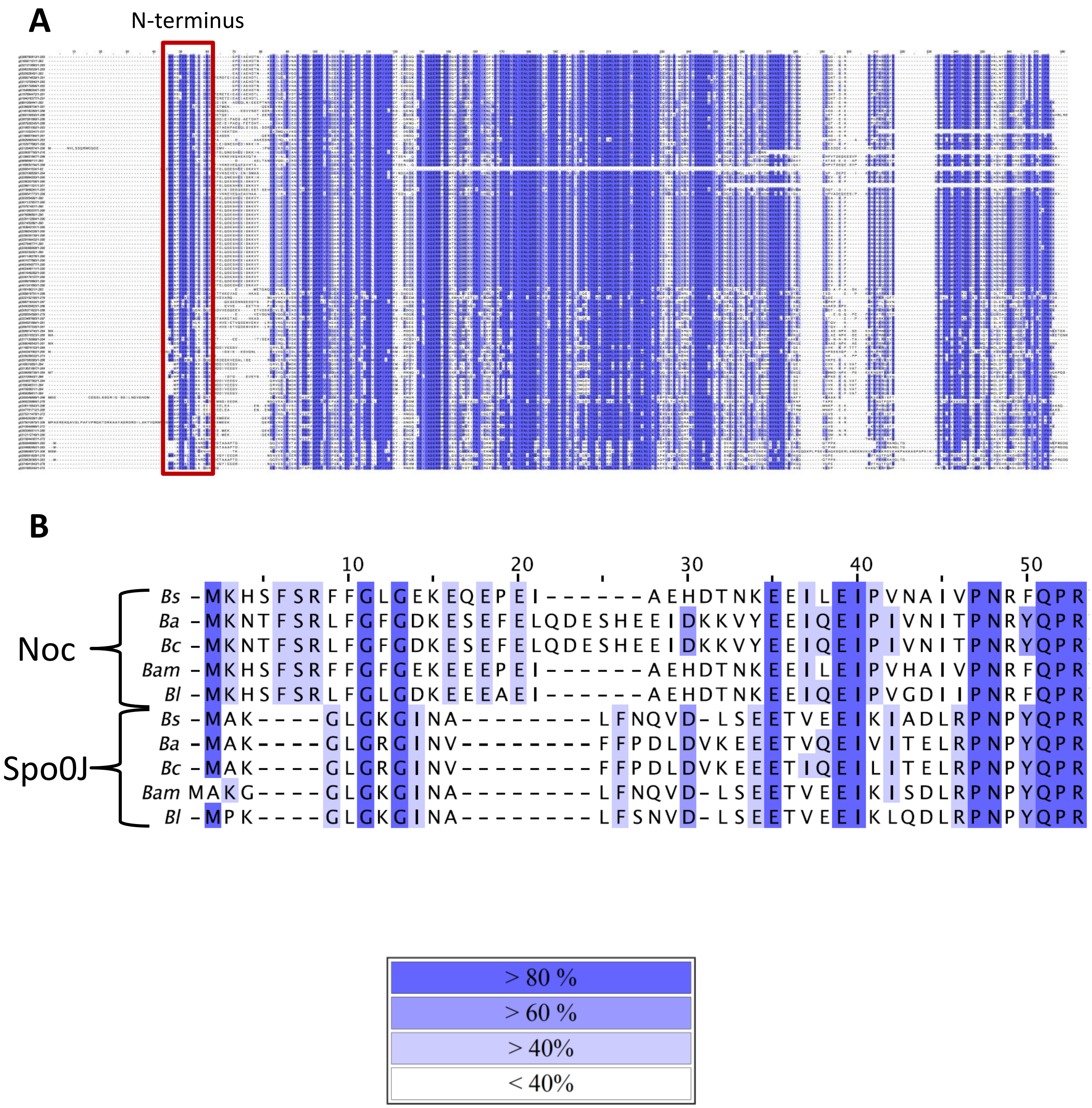
A
N-terminus
B
10
20
30
40
50
Noc
Spo0J
Bs -MKHSFSRFFGLGEKEQEPEI-------AEHDTNKEEILEIPVNAIVPNRFQPR
Ba -MKNTFSRLFGFGDKESEFELQDESHEEIDKKVYEEIQEIPIVNITPNRYQPR
Bc -MKNTFSRLFGFGDKESEFELQDESHEEIDKKVYEEIQEIPIVNITPNRYQPR
Bam -MKHSFSRFFGFGEKEEEPEI-------AEHDTNKEEILEIPVHAIVPNRFQPR
Bl -MKHSFSRLFGLGDKEEEAEI-------AEHDTNKEEIQEIPVGDIIPNRFQPR
Bs -MAK----GLGKGINA---------LFNQVD-LSEETVEEIKIADLRPNPYQPR
Ba -MAK----GLGRGINV---------FFPDLDVKEEETVQEIVITELRPNPYQPR
Bc -MAK----GLGRGINV---------FFPDLDVKEEETIQEILITELRPNPYQPR
Bam MAKG----GLGKGINA---------LFNQVD-LSEETVEEIKISDLRPNPYQPR
Bl -MPK----GLGKGINA---------LFSNVD-LSEETVEEIKLQDLRPNPYQPR
> 80 %
> 60 %
> 40%
< 40%

Supplementary Figure S3

Size-exclusion chromatography of purified Noc, and NocNΔ10 and NocCΔ50 truncated proteins. Proteins were run on a calibrated Superdex 200 10/300 GL gel filtration column (GE Healthcare) and approximate molecular weights were estimated by comparison to a set of known protein standards (See Supplementary Methods). The apparent molecular weights were as follows with the theoretical values in brackets: Noc 162.3 kDa (monomer, 32.8 kDa; dimer, 65.6 kDa; tetramer, 131.2 kDa), NocNΔ10 172.6 kDa (monomer, 31.6 kDa; dimer, 63.2 kDa; tetramer, 126.4 kDa) and NocCΔ50 38.9 kDa (monomer, 26.9 kDa; dimer, 53.8 kDa). The standard curve used to calibrate the column is shown below the elution profile.

Figure S3

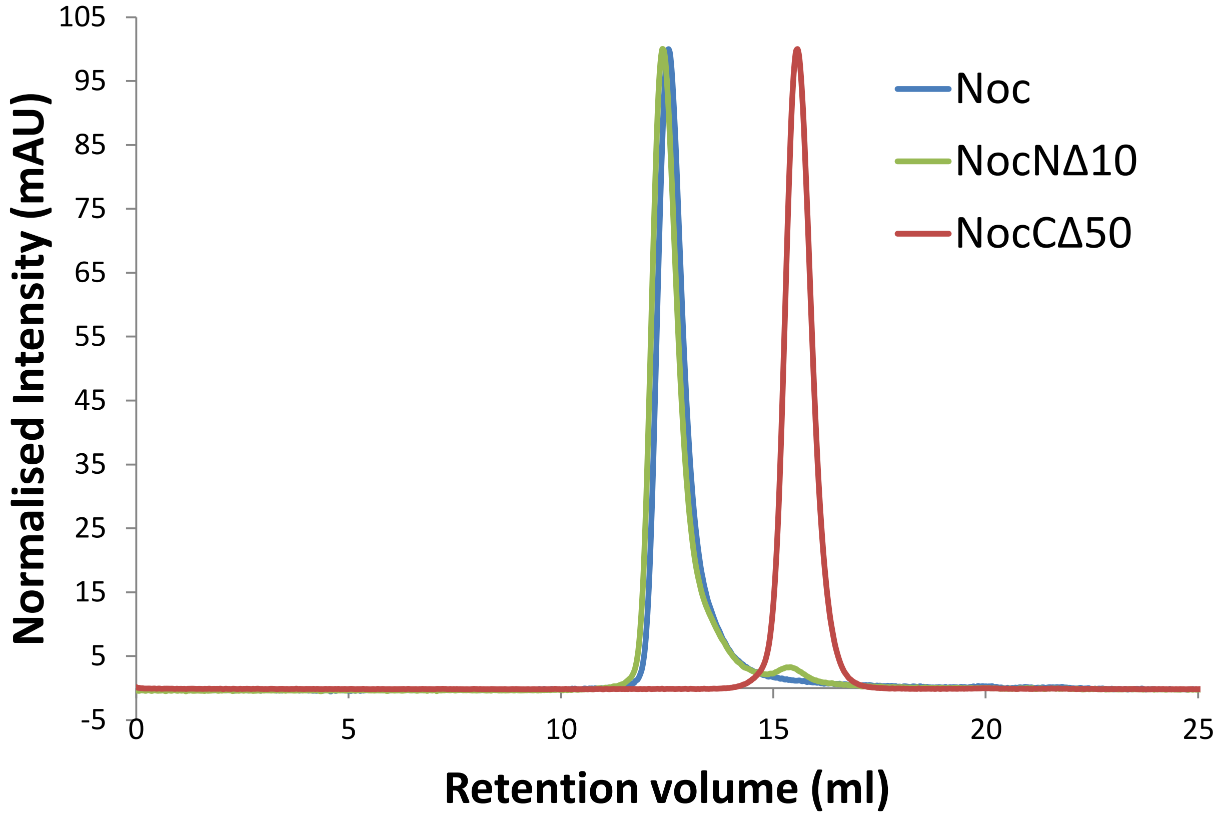

Normalised Intensity (mAU)
105
95
85
75
65
55
45
35
25
15
5
-5
0
5
10
15
20
25
Retention volume (ml)
Noc
NocNΔ10
NocCΔ50

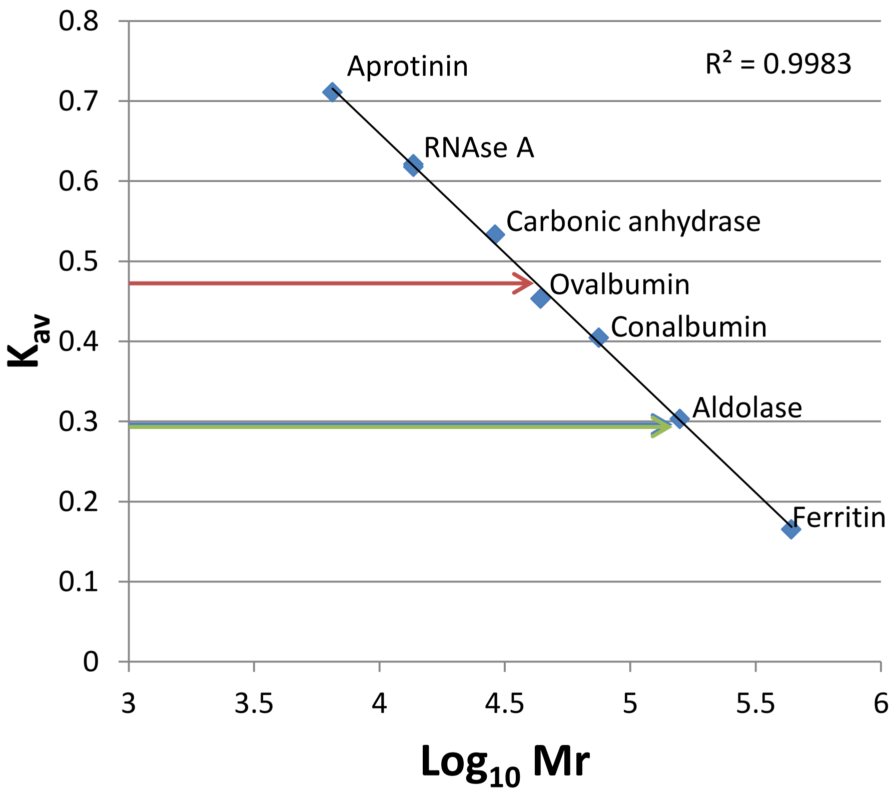

K_{av}
0.8
0.7
0.6
0.5
0.4
0.3
0.2
0.1
0
3
3.5
4
4.5
5
5.5
6
Log_{10} Mr
Aprotinin
RNAse A
Carbonic anhydrase
Ovalbumin
Conalbumin
Aldolase
Ferritin
R^2 = 0.9983

Supplementary Figure S4

(**A**) Histogram showing the effects of overproducing Noc mutants on cell length. Exponentially growing cells of strains 168 (WT), DWA117 (Δ*noc*), 119 (Δ*noc*, $P_{spac(hy)}$-*noc*), 282 (Δ*noc*, $P_{spac(hy)}$-*noc*NΔ10), 226 (Δ*noc*, $P_{spac(hy)}$-*noc*F5E) and 227 (Δ*noc*, $P_{spac(hy)}$-*noc*R7E) were examined after growth for 90 min in the presence of 1 mM IPTG. Cell membranes were stained with FM5-95 and used to measure cell length (WT, *n* =322; Δ*noc*, *n* =508; Noc, *n* =395; NΔ10, *n* =551; F5E, *n* =505; R7E, *n* =484).

(**B**) Effects of Noc mutants on sporulation. Strains DWA119 (Δ*noc*, $P_{spac(hy)}$-*noc*), 343 (Δ*noc*, $P_{spac(hy)}$-*noc*ΔK2), 344 (Δ*noc*, $P_{spac(hy)}$-*noc*K2A), 226 (Δ*noc*, $P_{spac(hy)}$-*noc*F5E), 346 (Δ*noc*, $P_{spac(hy)}$- *noc*ΔF5, S6), 347 (Δ*noc*, $P_{spac(hy)}$-*noc*R7A), 227 (Δ*noc*, $P_{spac(hy)}$-*noc*R7E) and 348 (Δ*noc*, $P_{spac(hy)}$-*noc*K14A) were grown on nutrient agar plates in the absence and presence of 1 mM IPTG, as indicated. Plates were photographed after growth for 48 h at 37 °C.

Figure S4

A

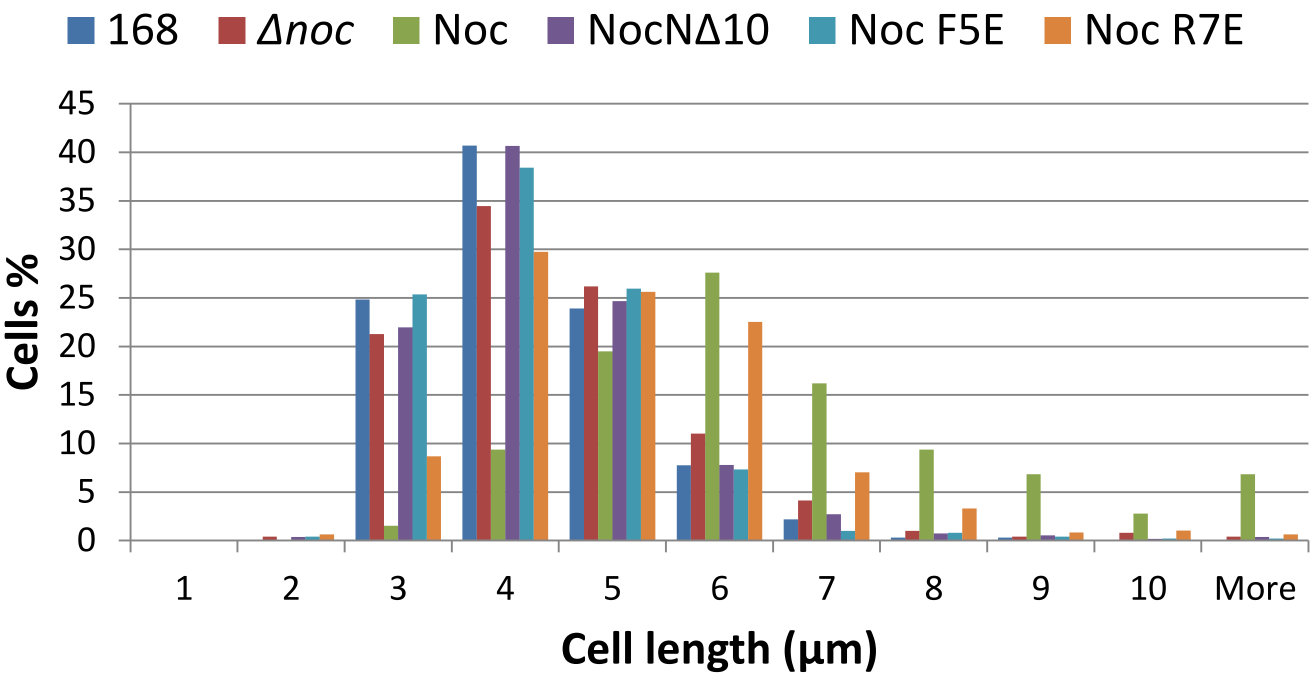
168
Δnoc
Noc
NocNΔ10
Noc F5E
Noc R7E
45
40
35
30
25
20
15
10
5
0
Cells %
1
2
3
4
5
6
7
8
9
10
More
Cell length (µm)

B

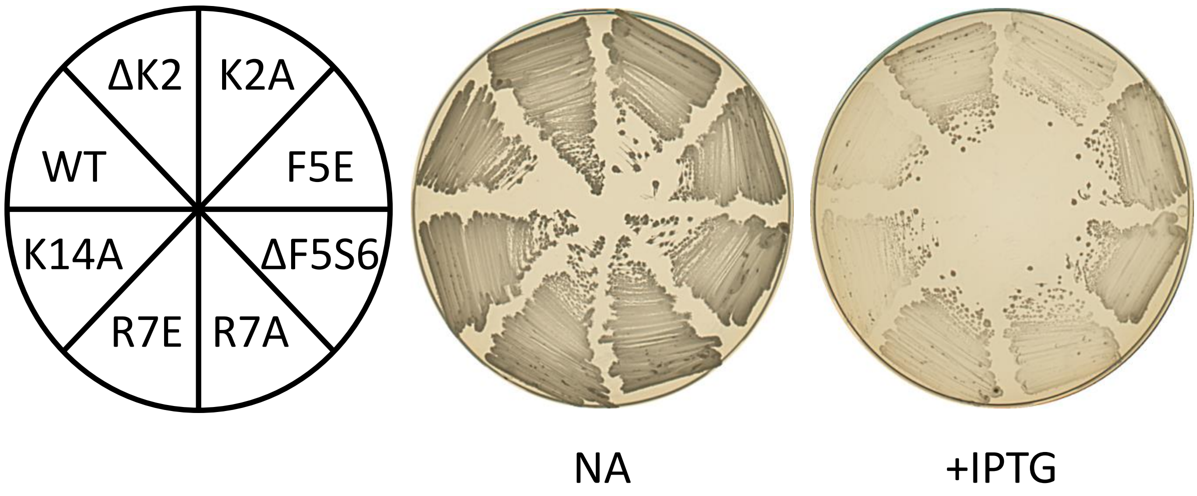
ΔK2
K2A
WT
F5E
K14A
ΔF5S6
R7E
R7A
NA
+IPTG

Supplementary Figure S5

(**A**) ^{HCV}AH-GFP is unable to rescue to the growth defect of Δ*noc* Δ*minCD* at 39 °C. Strains DWA564 (Noc-mYFP) and DWA629 (^{HCV}AH-GFP) were streaked on plates with either no additions (NA) or 0.5 % w/v xylose, as indicated, and photographed after incubation for 18 h at 39 °C.

(**B**) ^{HCV}AH-NocNΔ10-YFP rescues the growth defect of Δ*noc* Δ*minCD* at 39 °C. Strains DWA564 (Noc-mYFP) and DWA636 (^{HCV}AH-NocNΔ10-YFP) were streaked on plates with either no additions (NA) or 0.5 % w/v xylose, as indicated, and photographed after incubation for 18 h at 39 °C.

(**C**) TM-NocNΔ10 is unable to rescue to the growth defect of Δ*noc* Δ*minCD* at 39 °C. Strains DWA564 (Noc-mYFP) and DWA562 (TM-NocNΔ10-mYFP) were streaked on plates with either no additions (NA) or 0.5 % w/v xylose, as indicated, and photographed after incubation for 18 h at 39 °C.

(**D**) Production of TM-NocNΔ10 often leads to multiple defects in chromosome segregation. Exponentially growing cells of strain DWA548 (Δ*noc,* P_{xyl}-TM-*noc*NΔ10-*myfp*) were cultured in the presence of 1 % w/v xylose and observed 2 h post-induction. Arrow-heads indicate some of the DNA-damage events. Cell membranes and DNA were stained with FM5-95 and DAPI, respectively. The insets show the corresponding phase contrast images. Scale bar = 5 µm.

Figure S5

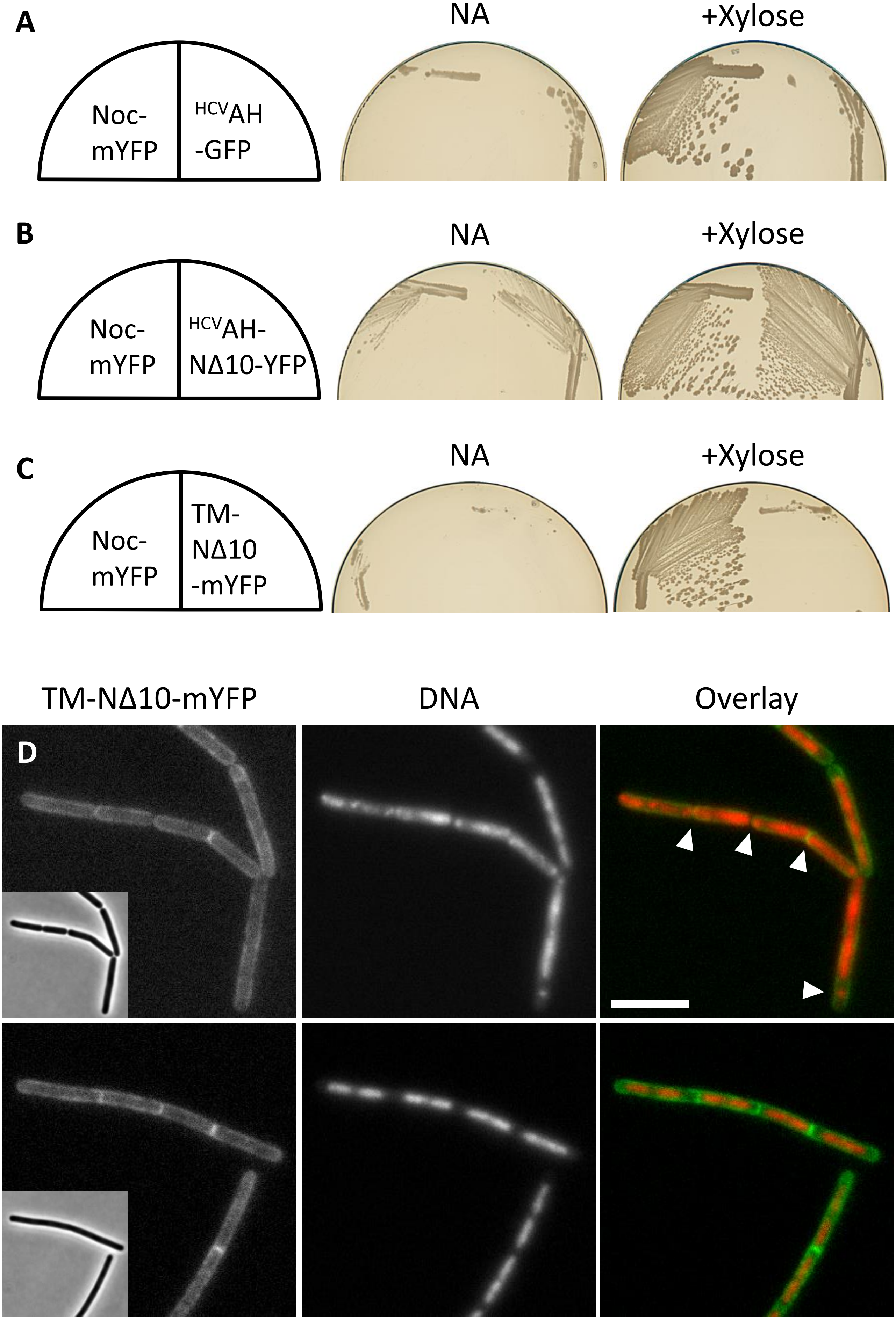
A
NA
+Xylose
Noc-
mYFP
HCVAH
-GFP
B
NA
+Xylose
Noc-
mYFP
HCVAH-
NΔ10-YFP
C
NA
+Xylose
Noc-
mYFP
TM-
NΔ10
-mYFP
TM-NΔ10-mYFP
DNA
Overlay
D

Supplementary Figure S6

(**A**-**B**) Localisation of GFP with a JunLZ dimerisation domain fused to either the amphipathic helix of *E. coli* MinD (pTS37) (Positive-control) (**A**), or the N-terminal 30 amino acids of Noc (pDWA20) (**B**). Constructs were expressed in *E. coli* DH5α by the inclusion of 0.2 % w/v arabinose in the media.

(**C**) Localisation in *B. subtilis* cells of a fusion between the N-terminal 50 amino acids of Noc and mYFP (Strain DWA225). The cartoons below each panel show a schematic of the relevant product.

(**D**) Western blots showing that the GFP-JunLZ-MinD (35 kDa) and GFP-JunLZ-Noc30 (36 kDa) fusions are intact and produced at comparable levels in *E. coli*. Exponentially growing cells were grown with (+) and without (-) induction (0.2 % w/v arabinose), as indicated, for 60 min at 37 °C, before being recovered and processed. Primary antibodies were used at a dilution of 1:20,000 (αNoc) and 1:10,000 (αGFP). The * denotes a non-specific band. The ** denotes limited degradation of the GFP-JunLZ-Noc30 protein.

(**E**) Western blots showing that the intact Noc50-mYFP (35.4 kDa) fusion is produced in *B. subtilis* (DWA215). Total protein from strains 168 (WT) and DWA117 (Δ*noc*) were included on the blot to facilitate size comparisons; Noc, 32.8 kDa. Exponentially growing cells were grown with induction (0.5 % w/v xylose) for 60 min at 37 °C, before being recovered and processed. Primary antibodies were used at a dilution of 1:20,000 (αNoc) and 1:10,000 (αGFP).

Figure S6

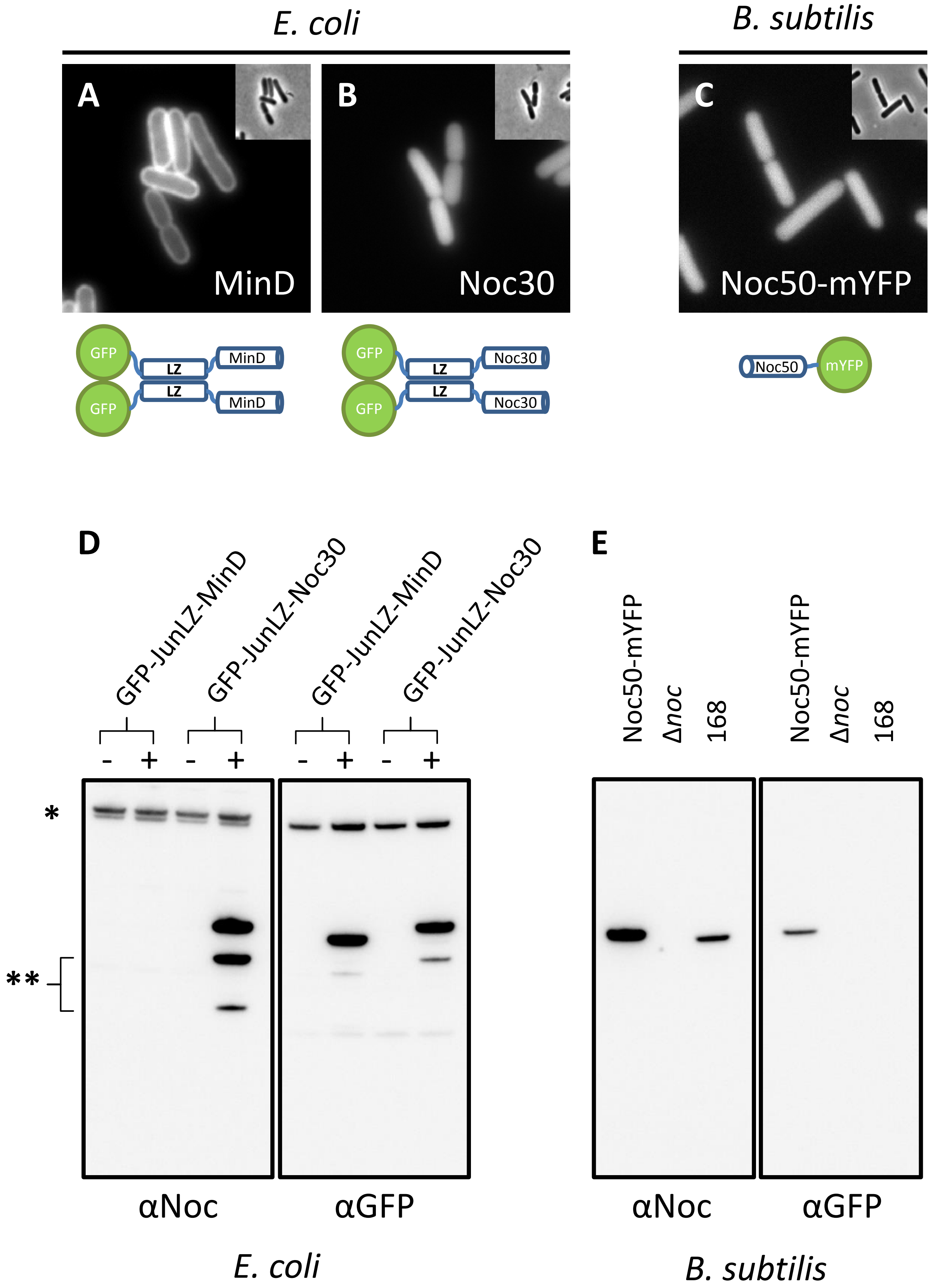
E. coli
B. subtilis
A
MinD
B
Noc30
C
Noc50-mYFP
GFP
LZ
MinD
Noc30
Noc50
mYFP
D
GFP-JunLZ-MinD
GFP-JunLZ-Noc30
GFP-JunLZ-MinD
GFP-JunLZ-Noc30
- + - + - + - +
*
**
αNoc
αGFP
E. coli
E
Noc50-mYFP
Δnoc
168
Noc50-mYFP
Δnoc
168
αNoc
αGFP
B. subtilis

Supplementary Figure S7

(**A**) Multiple sequence alignment of Noc and Spo0J homologues showing the conserved ParB boxes. Reference sequences were aligned using Clustal Omega. The figure was prepared using Jalview and is coloured according to percentage identity, the colour scheme is the same as in Supplementary FigS2. Species abbreviations: *B. subtilis*, ***Bs***; *B. anthracis*, ***Ba***; *B. cereus*, ***Bc***; *B. amyloliquefaciens*, ***Bam***; *B. licheniformis*, ***Bl***. The Noc residues examined in this study are highlighted in red and labelled, with the equivalent *B. subtilis* Spo0J residue number shown below in brackets.

(**B**) Non-functional ParB-box mutants are dominant-negative. Overproduction of Noc Q68R, G86S, R88A or R89A in a *noc*+ Δ*minCD* background prevents growth at 42 °C but not at 30 °C. Strains encoding the indicated $P_{spac(hy)}$- driven *noc* alleles were streaked on plates containing 1 mM IPTG and incubated at the indicated temperature for 18 h before being photographed. Strains used: DWA362 (WT Noc), DWA363 (NΔ10), DWA364 (Q68R), DWA365 (G86S), DWA559 (R88A), DWA560 (R89A), and DWA561 (R91A).

(**C**) Western blots showing the relative levels of Noc proteins (αNoc) produced under the control of the $P_{spac(hy)}$ promoter. All proteins are intact and are expressed at comparable levels. FtsZ levels are shown to control for sample loading. Exponentially growing cells were grown with inducer (1 mM IPTG) for 60 min at 37 °C, before being recovered and processed. Primary antibodies were used at a dilution of 1:20,000 (αNoc) and 1:10,000 (αFtsZ). Strains used: 168 (WT parent), DWA117 (Δ*noc*), DWA119 (Noc), DWA282 (NΔ10), DWA283 (Q68R), DWA284 (G86S), DWA551 (R88A), DWA552 (R89A) and DWA553 (R91A).

(**D**-**G**) Effect of Noc overproduction ($P_{spac(hy)}$-*noc*) on the localisation of NocNΔ10-YFP and NocG86S-mYFP. Cells of strains DWA370 (NocNΔ10-YFP) and DWA371 (NocG86S-mYFP) were examined after growth for 1 h in the absence (**D** and **E**) and presence (**F** and **G**) of 1 mM IPTG. Xylose (0.5 % w/v) was included to induce the expression of the YFP fusions. Insets show the corresponding phase contrast images. Scale bar = 5 µm.

Figure S7

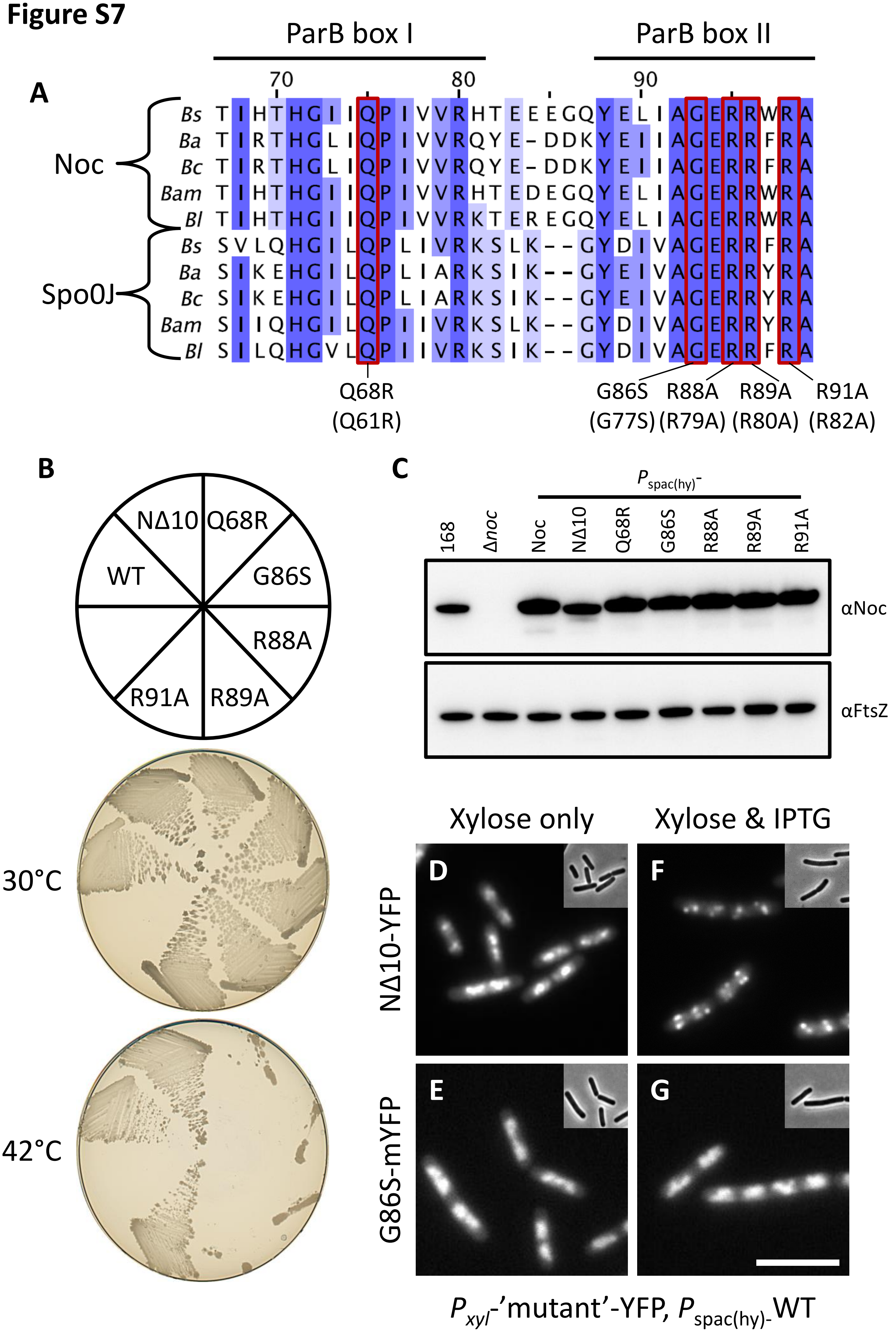

ParB box I
ParB box II
70
80
90
A
Noc
Spo0J
Bs
Ba
Bc
Bam
Bl
Q68R
(Q61R)
G86S
(G77S)
R88A
(R79A)
R89A
(R80A)
R91A
(R82A)
B
NΔ10
Q68R
WT
G86S
R88A
R91A
R89A
30°C
42°C
C
Pspac(hy)-
168
Δnoc
Noc
NΔ10
Q68R
G86S
R88A
R89A
R91A
αNoc
αFtsZ
Xylose only
Xylose & IPTG
D
F
E
G
NΔ10-YFP
G86S-mYFP
Pxyl-'mutant'-YFP, Pspac(hy)-WT

Supplementary Figure S8

Strains DWA78 (P_{spac}-*noc* + pSG4929) (**A** and **B**) and DWA429 (P_{spac}-*noc* + pDWA117) (**C** and **D**) were grown in LB at 37 °C and examined after growth for 90 min either without further additions (**A** and **C**) or after induction with 0.1 mM IPTG (**B** and **D**). Cell membranes and DNA were stained with FM5-95 and DAPI, respectively. Insets show the corresponding phase contrast images. Scale bar = 5 µm.

(**E**) Toxicity of Noc variants in the presence of the NBS plasmid pSG4929. Strains carrying pSG4929 and encoding the indicated $P_{spac(hy)}$- driven *noc* alleles were streaked on plates with either no additions (NA) or containing 1 mM IPTG, as indicated. Strains used: DWA523 (WT), DWA524 (NΔ10), DWA525 (Q68R), DWA526 (G86S), DWA623 (R88A), DWA624 (R89A), and DWA625 (R91A). Plates were incubated at 37 °C for 18 h before being photographed.

Figure S8

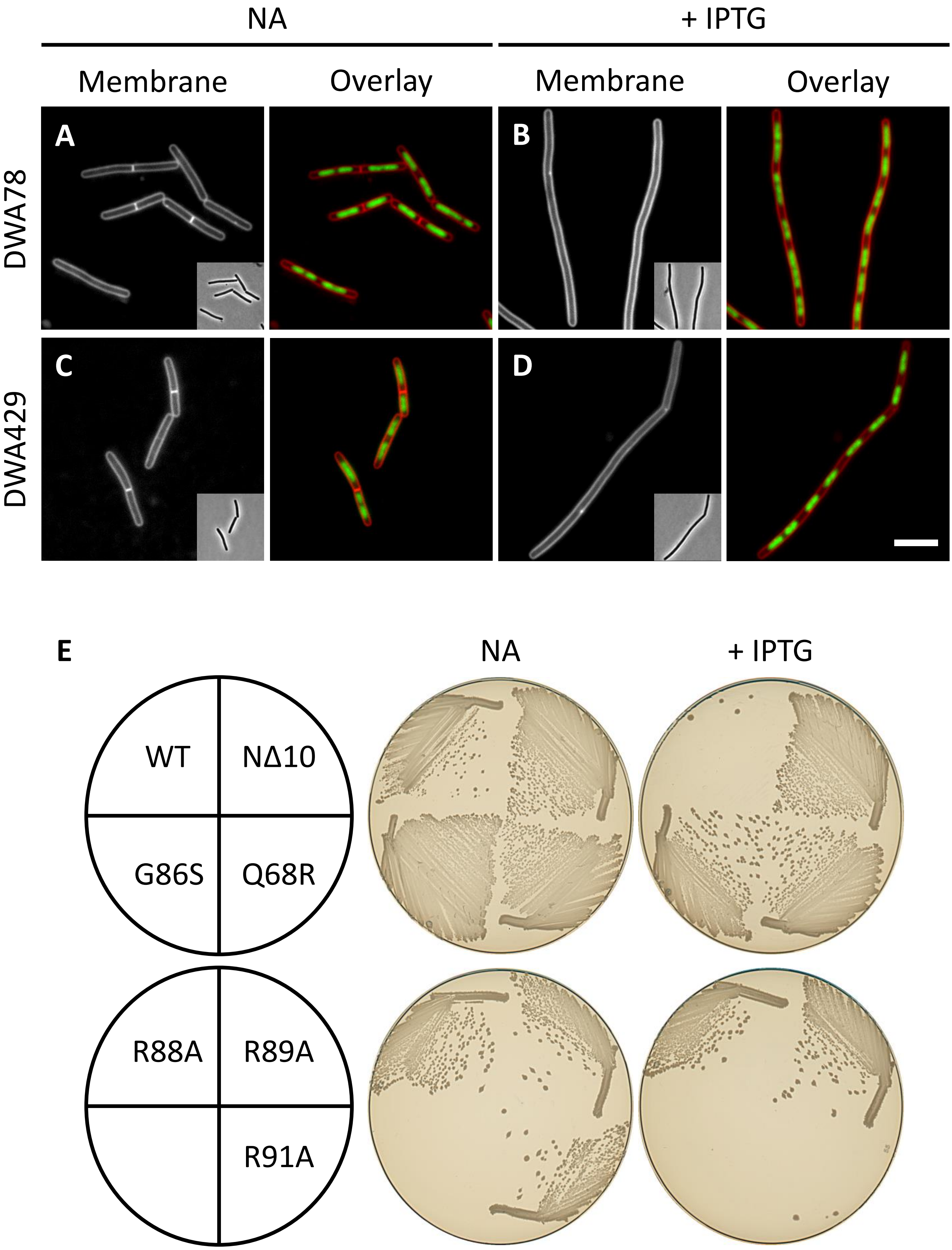
NA
+ IPTG
Membrane
Overlay
Membrane
Overlay
A
B
C
D
DWA78
DWA429
E
NA
+ IPTG
WT
NΔ10
G86S
Q68R
R88A
R89A
R91A

Supplementary Figure S9

Western blots using αNoc (**A**) or αSpo0J (**B**) showing the relative levels of the indicated proteins produced in *E. coli* MG1655. Exponentially growing cells were grown with induction (0.5 mM IPTG) for 30 min at 37 °C, before being recovered and processed. Primary antibodies were used at a dilution of 1:20,000. Strains used: DWA260 (Empty vector), DWA261 (Noc), DWA266 (NocNΔ10), DWA267 (NocCΔ50), DWA271 (Spo0J) and DWA272 (Noc-Spo0J). Lanes labelled with an asterisk contain proteins that were not used in this study.

Figure S9

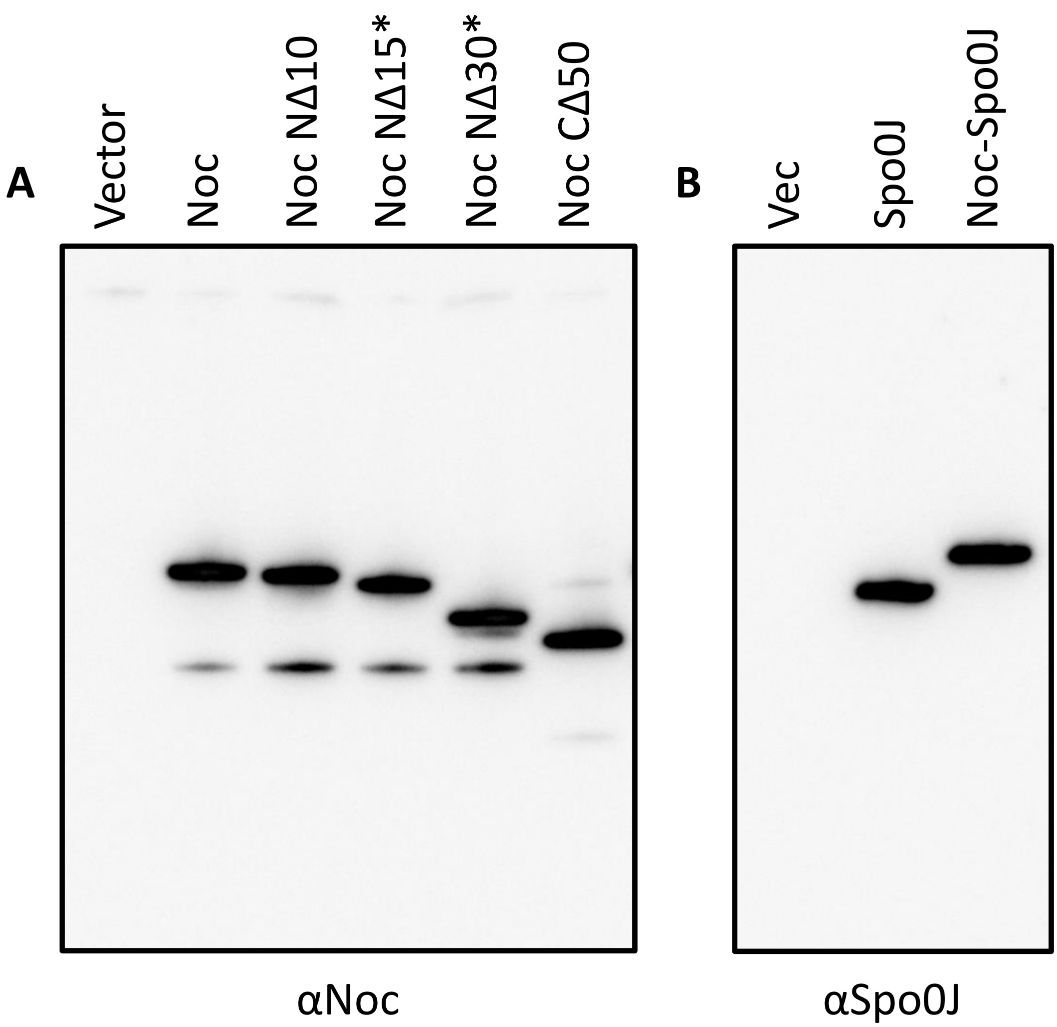
A
Vector
Noc
Noc NΔ10
Noc NΔ15*
Noc NΔ30*
Noc CΔ50
αNoc
B
Vec
Spo0J
Noc-Spo0J
αSpo0J

Supplementary Figure S10

Cells of strains DWA334 (MC1000 parent), DWA337 (Δ*sulA*), DWA335 (Δ*slmA*) and DWA336 (Δ*minCD*) carrying plasmid pDWA37 ($P_{A1/O4/O3}$-*noc*) were examined after growth for 1 h in the absence (**A**, **C**, **E**, and **G**) and presence (**B**, **D, F,** and **H**) of 1 mM IPTG. Cell membranes and DNA were stained with FM5-95 and DAPI, respectively. Insets show the corresponding phase contrast images. Scale bar = 5 µm.

Figure S10

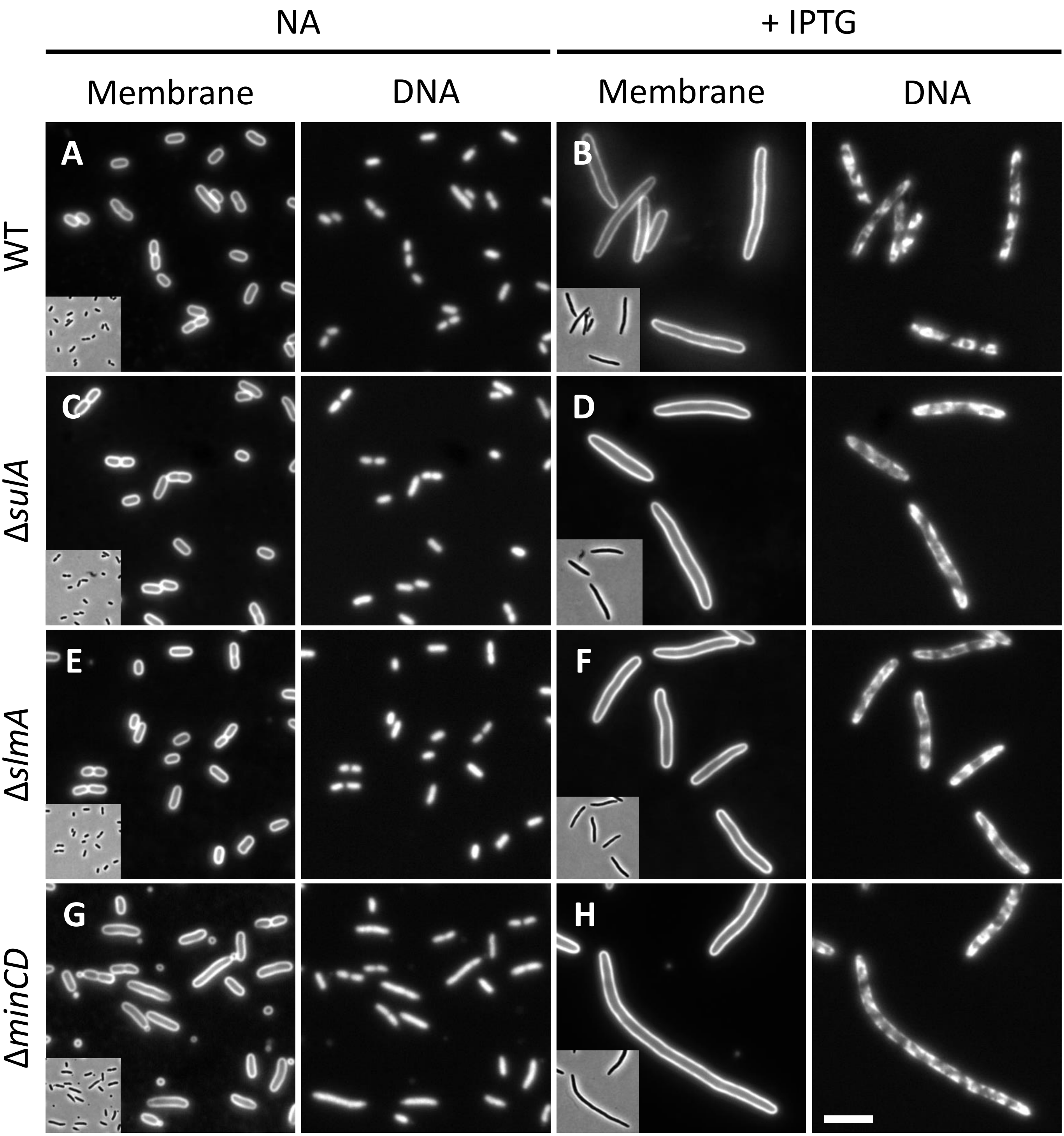

NA
+ IPTG
Membrane
DNA
Membrane
DNA
A
B
C
D
E
F
G
H
WT
ΔsulA
ΔslmA
ΔminCD

Supplementary Figure S11

Western blots showing the relative levels of Noc-YFP fusions (αNoc). All fusion proteins are intact and are produced at comparable levels (except S4L/G86S). FtsZ levels are shown to control for sample loading. Exponentially growing cells were grown with inducer (0.5 % w/v xylose) for 120 min at 37 °C, before being recovered and processed. Primary antibodies were used at a dilution of 1:20,000 (αNoc) and 1:10,000 (αFtsZ). Strains used: DWA206 (WT Noc-mYFP), DWA211 (F5E-YFP), DWA212 (R7E-YFP), DWA285 (Q68R-mYFP), DWA286 (G86S-mYFP), DWA316 (K2E-mYFP), DWA318 (F9E-mYFP), DWA322 (F5A-mYFP), DWA323 (F8A-mYFP), DWA325 (F9A-mYFP), DWA328 (S4A-mYFP), DWA329 (S4L-mYFP), DWA349 (S4L/G86S-mYFP), DWA382 (NΔ10-mYFP), DWA545 (R88A-mYFP), DWA546 (R89A-mYFP), DWA547 (R91A-mYFP), DWA606 (K164A-mYFP) and DWA634 (K14A-YFP).

Figure S11

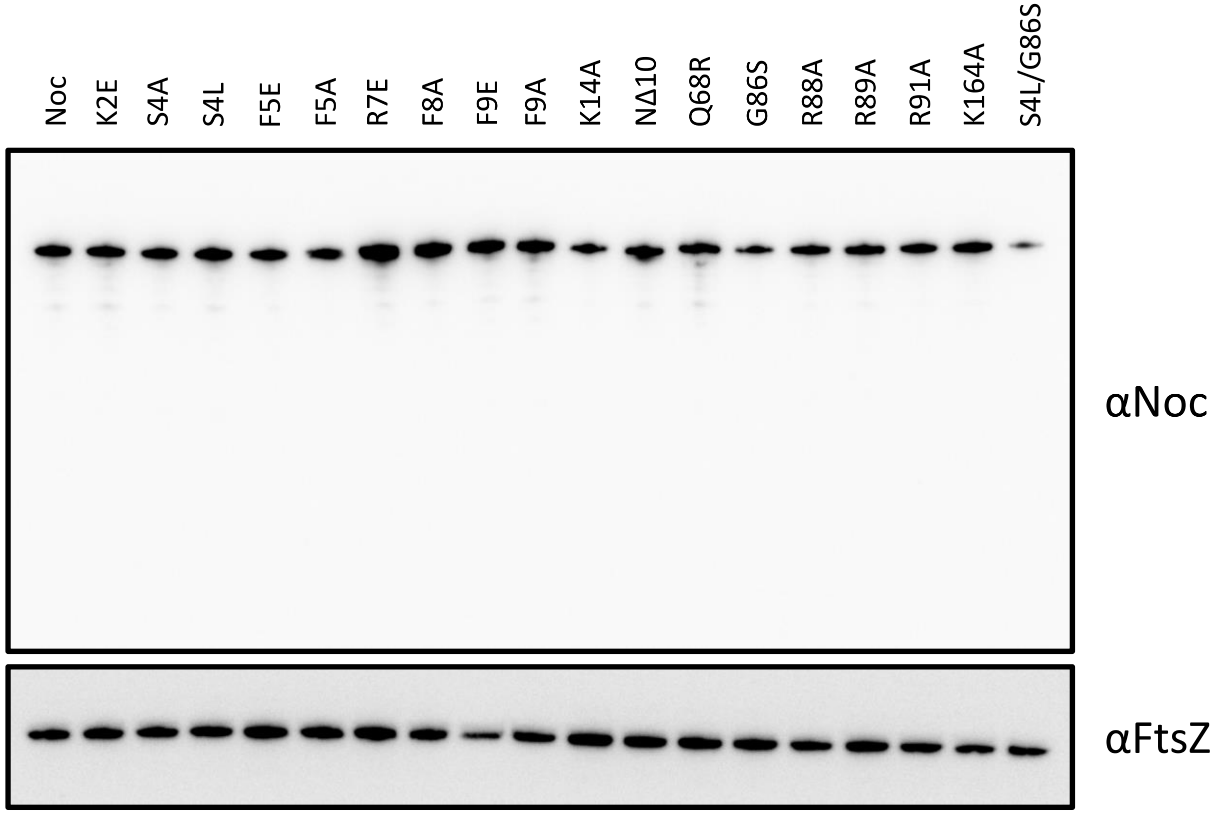
Noc
K2E
S4A
S4L
F5E
F5A
R7E
F8A
F9E
F9A
K14A
NΔ10
Q68R
G86S
R88A
R89A
R91A
K164A
S4L/G86S
αNoc
αFtsZ

Movie legends

Movies 1-3

Time-lapses showing localisation of Noc-mYFP (**Movies 1** and **2**) and NocNΔ10-mYFP (**Movie 3**) in strains DWA206 and 382 using TIRF microscopy. Cells were grown in CH + 0.5 % (w/v) xylose and examined either without further additions (**Movies 1** and **3**) or after treatment for 5 min with 100 µM CCCP (**Movie 2**). TIRF images were acquired automatically every 500 ms for *c.a.* 30 s. Images were scaled identically and the corresponding 'zero-angle' images are shown above each panel. Note the absence of NocNΔ10-mYFP signal from the cell surface in Movie 3.

Movies 4 and 5

Time-lapses showing localisation of plasmid DNA (TetR-mCherry) in strain DWA429 (P_{xyl}-*tetR-mCherry*, P_{spac}-*noc* + pDWA117). Cells were grown in competence medium + 0.5 % (w/v) xylose and examined either without further additions (**Movie 4**) or after induction for 2 hours with 1 mM IPTG (**Movie 5**). Images were acquired automatically every 10 s for 60 s. The corresponding phase-contrast image is shown above each panel. Scale bars = 5 µm.

Supplementary References

Christensen-Dalsgaard M, Overgaard M, Winther KS, Gerdes K (2008) RNA decay by messenger RNA interferases. *Methods in enzymology* **447:** 521-535

Daniel RA, Harry EJ, Errington J (2000) Role of penicillin-binding protein PBP 2B in assembly and functioning of the division machinery of *Bacillus subtilis*. *Mol Microbiol* **35:** 299-311

Ebersbach G, Galli E, Moller-Jensen J, Lowe J, Gerdes K (2008) Novel coiled-coil cell division factor ZapB stimulates Z ring assembly and cell division. *Mol Microbiol* **68:** 720-735

Hicks DB, Cohen DM, Krulwich TA (1994) Reconstitution of energy-linked activities of the solubilized F1F0 ATP synthase from Bacillus subtilis. *Journal of bacteriology* **176:** 4192-4195

Landgraf D, Okumus B, Chien P, Baker TA, Paulsson J (2012) Segregation of molecules at cell division reveals native protein localization. *Nature methods* **9:** 480-482

Lau IF, Filipe SR, Søballe B, Økstad OA, Barre FX, Sherratt DJ (2003) Spatial and temporal organization of replicating *Escherichia coli* chromosomes. *Mol Microbiol* **49:** 731-743

Lin DC, Grossman AD (1998) Identification and characterization of a bacterial chromosome partitioning site. *Cell* **92:** 675-685

Lucet I, Feucht A, Yudkin MD, Errington J (2000) Direct interaction between the cell division protein FtsZ and the cell differentiation protein SpoIIE. *EMBO J* **19:** 1467-1475

Nyholm TK, Özdirekcan S, Killian JA (2007) How protein transmembrane segments sense the lipid environment. *Biochemistry* **46:** 1457-1465

Quisel JD, Burkholder WF, Grossman AD (2001) *In vivo* effects of sporulation kinases on mutant Spo0A proteins in *Bacillus subtilis*. *Journal of bacteriology* **183:** 6573-6578

Santana M, Ionescu MS, Vertes A, Longin R, Kunst F, Danchin A, Glaser P (1994) Bacillus subtilis F0F1 ATPase: DNA sequence of the atp operon and characterization of atp mutants. *Journal of bacteriology* **176:** 6802-6811

Scholefield G, Errington J, Murray H (2012) Soj/ParA stalls DNA replication by inhibiting helix formation of the initiator protein DnaA. *EMBO J* **31:** 1542-1555

Strahl H, Hamoen LW (2010) Membrane potential is important for bacterial cell division. *Proc Natl Acad Sci U S A* **107:** 12281-12286

Szeto TH, Rowland SL, Habrukowich CL, King GF (2003) The MinD membrane targeting sequence is a transplantable lipid-binding helix. *The Journal of biological chemistry* **278:** 40050-40056

Wu LJ, Errington J (2004) Coordination of cell division and chromosome segregation by a nucleoid occlusion protein in *Bacillus subtilis*. *Cell* **117:** 915-925

Wu LJ, Ishikawa S, Kawai Y, Oshima T, Ogasawara N, Errington J (2009) Noc protein binds to specific DNA sequences to coordinate cell division with chromosome segregation. *EMBO J* **28:** 1940-1952
