# Supplementary material for: Nucleoid occlusion protein Noc recruits DNA to the bacterial cell membrane
Source: EMBO J. 2015 Jan 7;34(4):491–501. doi: 10.15252/embj.201490177 (PMC4331003; doi:10.15252/embj.201490177)
Supplement: Supplementary file 7 [file embj0034-0491-sd7.pdf]

THE
EMBO
JOURNAL

Manuscript EMBO-2014-90177

Nucleoid Occlusion protein Noc recruits DNA to the Bacterial Cell Membrane

David William Adams, Ling Juan Wu and Jeff Errington

Corresponding author: Jeff Errington, Newcastle University

Review timeline:

Submission date:	27 September 2014
Editorial Decision:	30 October 2014
Revision received:	25 November 2014
Accepted:	05 December 2014

Transaction Report:

(Note: With the exception of the correction of typographical or spelling errors that could be a source of ambiguity, letters and reports are not edited. The original formatting of letters and referee reports may not be reflected in this compilation.)

Editor: David del Alamo

1st Editorial Decision 30 October 2014

Thank you for the submission of your manuscript entitled "Nucleoid Occlusion protein Noc recruits DNA to the Bacterial Cell Membrane". We have now received the full set of reports from the referees, which I copy below.

As you can see from their comments, all three referees enthusiastically support the publication of your study in The EMBO Journal, but point out to a few concerns, mainly related to specific technical issues and certain clarifications, that will require your attention before we can further proceed with the acceptance of your manuscript.

Given these positive opinions from the referees, I would like to invite you to submit a revised version of you work. We normally allow a single round of major revision only, which should be submitted within the next three months. Should you foresee a problem in meeting the three-month deadline, please let us know in advance and we may be able to grant an extension. Similarly, do not hesitate to contact me if you have any questions, need further input on the referee comments or you anticipate any problems along the revision process.

Thank you very much again for the opportunity to consider your work for publication. I look forward to your revision.

Referee #1:

This paper examines the mechanism by which Noc inhibits cell division in Bacillus subtilis. Previous work has shown that Noc appears to inhibit the assembly of the division machinery in the vicinity of DNA and binds DNA to function. It coordinates chromosome segregation with division as it binds DNA at specific palindromic binding sites that are absent in DNA surrounding the terminus region. However, little was known about how Noc specifically inhibits division as Noc does not appear to interact with any known division proteins. Here, based on a combination of genetic, biochemical and localization experiments in B. subtilis and an heterologous E. coli system, the authors propose that Noc inhibits cell division simply by connecting the DNA to the membrane, physically occluding the assembly of the division machinery.

The experiments are technically sound, thorough and convincing. The paper is clearly written, and the proposed mechanism is novel and attractive in its simplicity. I only have minor suggestions for improvement.

All of the fusion proteins used for the localization experiments are expressed from an inducible promoter. It would be reassuring to see that native expression of a Noc-mYFP fusion show similar localization pattern. This should be a straightforward experiment given the genetic tractability of B. subtilis. Perhaps this experiment has been done before, in which case it should be stated.

The images were generally effective in making the authors' points, except perhaps for Figure 5. The difference between panel B and C was not obvious. A higher magnification or quantification might help.

All movies should have a time stamp and calibration bar. Note that I did not understand the point about movie 3. What is it showing that the image does not show?

The title is correct (and safe) but not very informative about the proposed mechanism.

Referee #2:

The authors previously showed that the B.subtilis nucleoid occlusion (Noc) protein plays an important role in coupling assembly of the division apparatus at midcell to the location and replication/segregation status of the chromosome. Noc binds multiple NBS sites on the chromosome and interferes with division, but how it does so is still enigmatic.

Here, the authors show that Noc also binds the membrane via a weak N-terminal amphipathic helix, that binding of Noc to DNA and subsequent spreading/bridging is required for membrane-binding, and that both its chromosome-binding and membrane-binding activities are essential to its function. Noc also seems to recruit NBS-containing plasmids to the membrane in B.subtilis, and can also recruit the chromosome to the cell periphery when overexpressed to some undetermined level in E.coli. Interestingly, division is inhibited in both these cases, suggesting that drawing DNA close to the membrane can interfere with division in both organisms.

Based on these results, the authors propose a 'crowding' mechanism for Noc function, whereby the protein increases the density of nucleo-protein complexes near the membrane to a level sufficiently high to effectively interfere with assembly/stability of the division apparatus. More specific mechanisms are not excluded, however.

Overall, this study is an important contribution to the field, and the described properties of Noc will be of interest to a wider audience. However, parts of the study/presentation are incomplete in my opinion.

My critiques/comments are both of a technical (e.g. missing information on protein levels and integrity) and conceptual (especially point 14a-d, below) nature. This will require some additional work/documentation.

Comments:

1) CCCP sensitivity of Noc-YFP localzation.
I don't quite understand the effect of CCCP on Noc-YFP localzation, or the interpretation in the discussion (lines 345-347). If membrane-binding were delta-psi dependent (L 345), one would expect Noc-YFP to resemble delta10-Noc-YFP and localize to the nucleoid. In stead, the fusion is still in peripheral clusters that now no longer need be associated with the nucleoid. Why would CCCP affect DNA-binding of the full length Noc-YFP fusion?

2) Figure S1
The point of using the ATP synthase mutant (i.e. ATP-levels are less sensitive to CCCP) may escape a good chunk of readers. The rationale with some references should be explained in the text or the figure legend.

3) A key to how colour relates to percent identities in figures S2 and S7A is missing. Please include in legends.

4) Figure S4A
a) The panel is confusing without an explanation in the legend that you are measuring cell length after OVERproduction of Noc.
b) The panel is also incomplete without the length distributions of wt and noc- cells for comparison.
c) At what level of IPTG would Noc be expressed at normal level?

5) Figure S3 is incomplete as well.
a) What were the approximate MW's of Noc proteins?
b) How does this compare to the calculated MW's of monomer or dimer?
c) What were the MW standards, and were did they elute?

6) Table S1, functionality of mutants
a) Did authors do Westerns to ensure that mutants are not more (e.g. S4L) or less (all others) stable than wt?
b) What does 'More membrane' (S4L) mean?
c) The Table also seems incomplete. For example, several mutants (delta-K2, K2A, delta-F5S6, R7A, K14A) used in sporulation assays (Fig.S4B) are not included. Why not? It just seems odd that some mutants are used for some, but not all assays for Noc functionality.
d) Line 166. Why refer to the sporulation assay here, which is derivative at best? According to Table S1 it seems as if K2 and R7 actually do contribute to Noc function. K14A is not included in the table, but should.

7) Figure 3
The composition of the figure suggests that the YFP fusion to HCV-Noc was not functional.
a) Please discuss if the fusion was functional or not.
b) What were growth conditions for panels G and H?

8) Figure S5, TM-Noc
I'm somewhat surprised that the TM-Noc version does not seem to correct delta-Noc, delta-min at all.
a) Did you try this fusion at lower expression levels?
b) The results suggest the fusion should actually be toxic in wt or delta-noc cells at these levels of expression. Was it?
c) Does the TM-Noc fusion still not correct delta-Noc, delta-min at levels were it is not toxic to wt cells?
d) One might expect chromosome bisection (as in panel C) to results in an SOS response. However, cells still seem to be dividing. Perhaps the authors can comment on this?

9) Figure S6
A Western of the fusions that fail to accumulate on the membrane (panels B and C) needs to be included, to ensure the fusions are not simply degraded.

© European Molecular Biology Organization

10) Figure 4, spreading mutants
a) Again, the correction and transdominant experiments would be more convincing with a Western showing that all Noc variants are present at about the same level.
b) I would expect the delta-10 mutant to become transdominant at some level of overexpression. What is the level of overexpression (versus WT) in the transdominant assays approximately?
c) Line 239. Please indicate that these sporulation assays are not shown.

11) NBS plasmids
a) I assume that movies 4 and 5 are not TIRF microscopy? Wouldn't TIRF be more convincing if the point is to show more plasmid at the periphery of the cell?
b) Line 284. I'm actually more struck by the impression that the NBS plasmid now seems to prefer co-localization with nucleoids in the presence of Noc (i.e. it seems relatively depleted from internucleoid gaps, Figure 5C). Can the authors explain/comment on this?
c) Fig5E suggests that the delta-10 protein may cause plasmids to aggregate at poles. Did you look at the plasmid in this context? This might be consistent with DNA-bridging by Noc?

12) Expression in E.coli
a) It would be very helpful to know how much Noc protein is produced in these experiments, relative to say the normal level in B.subtilis. For example, a titration western with normalization to total protein or cell number could be added to figure S9.
b) Is Noc clustering/spreading still relevant at these levels of expression? Do the spreading mutants have any effect in E.coli?
c) Is the Noc-Spo hybrid functional in B.subtilis? I realize there are only few parS sites, but even if it has only partial 'Noc-like' activity, this would be of interest and might argue in favour of the model.

13) Plasmid construction
A better description of plasmids and details on their construction is needed.
For example, I'm not familiar with the PA1/04/03 promotor. Is this extra strong (see point 12, above)?

14) Model, figure 8
a) Does an NBS plasmid combined with fairly 'normal' levels of Noc cause division inhibition in E.coli as it does in B.subtilis? If so, you'd have a pretty good argument for the Noc-DNA 'crowding' model (though, see d below). If not, however, why not?
b) Does a parS plasmid combined with fairly 'normal' levels of the Noc-Spo hybrid cause division inhibition in either B.subtilis or E.coli. Again, if so, it would argue for the crowding model, rather than anything specific to Noc.
c) One conceptual problem I have with the crowding model is that the number of transertion-mediated contacts between the chromosome and the membrane is likely to be already so high in actively growing cells, that it is surprising if Noc-mediated contacts would make such a difference. I believe there are some numbers in the literature estimating the number of transertions at any one time. Maybe the authors can discuss this and the proposed contribution of Noc in a more quantitative fashion?
d) Lines 427-428. This 'natural ability' of the nucleoid to interfere with division may, of course, still depend on some other DNA-binding protein, which might interfere with the division apparatus in a specific way. The fact that such a factor has not yet been found in screens does not preclude this possibility. E.g. it could be DNA/RNA polymerase, or some other essential protein that can interfere with the division apparatus in an evolutionary conserved fashion, and relevant mutants may be difficult to generate/isolate. This may be worth pointing out to the reader.

15) Discussion, other points.
a) lines 361-362: Neither of the two statements is accurate, and they are also inherently inconsistent. If ParB-box mutants bind all over the chromosome, why wouldn't they bind plasmid DNA as well? The mutants may still prefer certain sequences on the chromosome, and whether or not they bind NBS plasmids was not really tested.
b) Line 376. This statement does not logically follow the previous sentence. The TM-Noc fusion can most likely still bind DNA as it does cause segregation problems.

c) Line 398. But, wouldn't the SOS response cause division inhibition due to chromosome breakage (see point 8)?

Referee #3:

This paper reports a comprehensive analysis of the Bacillus Noc, with surprising but convincing results. Noc was previously identified as a factor that inhibits FtsZ-ring assembly over the nucleoid, but the mechanism was not known. Multiple searches have failed to find any direct interaction of Noc with FtsZ, or with downstream Z-ring proteins. Here Adams, Wu and Errington discovered that Noc has an N-terminal amphipathic helix that binds it weakly to the membrane. This binding is essential for its inhibitory activity. Also essential is the ability of Noc to spread from the single NBS, forming a multivalent complex that can bind to the membrane more tightly than a single Noc. These points are nicely demonstrated and discussed as a novel regulatory mechanism. An especially strong experiment was the reconstitution of the membrane binding and division block in E. coli.
In the end I had expected to see some kind of mechanical model with road-blocks or barriers blocking advancing Z rings. I was pleased to see that the authors avoided this kind of detailed and premature speculation, and just left with the suggestion that the mass of nucleoid bound to the membrane somehow blocked the Z ring. A very nice study, important new discoveries and very well presented.

Minor editorial suggestion: Line 379 discusses the contrast of Noc mechanism to SlmA in E coli. This would be best to start as a new paragraph. Also, lines 388-392 would be better merged in that paragraph, leaving the new section to begin with "how does Noc inhibit.."

1st Revision - authors' response 25 November 2014

Referee #1:

This paper examines the mechanism by which Noc inhibits cell division in Bacillus subtilis. Previous work has shown that Noc appears to inhibit the assembly of the division machinery in the vicinity of DNA and binds DNA to function. It coordinates chromosome segregation with division as it binds DNA at specific palindromic binding sites that are absent in DNA surrounding the terminus region. However, little was known about how Noc specifically inhibits division as Noc does not appear to interact with any known division proteins. Here, based on a combination of genetic, biochemical and localization experiments in B. subtilis and an heterologous E. coli system, the authors propose that Noc inhibits cell division simply by connecting the DNA to the membrane, physically occluding the assembly of the division machinery.

The experiments are technically sound, thorough and convincing. The paper is clearly written, and the proposed mechanism is novel and attractive in its simplicity. I only have minor suggestions for improvement.

All of the fusion proteins used for the localization experiments are expressed from an inducible promoter. It would be reassuring to see that native expression of a Noc-mYFP fusion show similar localization pattern. This should be a straightforward experiment given the genetic tractability of B. subtilis. Perhaps this experiment has been done before, in which case it should be stated.

To check that the localisation pattern of Noc-mYFP is representative of native expression levels we examined cells induced with a 10-fold lower concentration of inducer (*i.e.* 0.05 % w/v xylose). The localisation was indistinguishable from that reported in the manuscript and we found no evidence that the localisation pattern varies with expression level. This is consistent with the functionality of the fusion protein at this low level of induction, as reported in Table S1. Moreover, given the agreement between the numerous *noc* mutants and the localisation of their respective fusion proteins characterised in this work, the data strongly support the idea that the localisation of the fusion is robust.

The images were generally effective in making the authors' points, except perhaps for Figure 5. The difference between panel B and C was not obvious. A higher magnification or quantification might help.

As requested, we have replaced the panels in Figure 5A, B and C with higher magnification images.

All movies should have a time stamp and calibration bar. Note that I did not understand the point about movie 3. What is it showing that the image does not show?

As requested, we have added the missing scale bars and added a time stamp for each movie. The TIRF microscopy in Movie 3 serves as an additional control for the epi-fluorescence images by demonstrating that NocN10-mYFP is absent from the cell surface. An explanatory note has been added to the legend to clarify this point.

The title is correct (and safe) but not very informative about the proposed mechanism.

Finding a suitable title can often prove challenging. We feel that the title communicates the key finding of the work to the readership without being overly speculative. We are of course open to editorial suggestions.

Referee #2:

The authors previously showed that the B.subtilis nucleoid occlusion (Noc) protein plays an important role in coupling assembly of the division apparatus at midcell to the location and replication/segregation status of the chromosome. Noc binds multiple NBS sites on the chromosome and interferes with division, but how it does so is still enigmatic.

Here, the authors show that Noc also binds the membrane via a weak N-terminal amphipathic helix, that binding of Noc to DNA and subsequent spreading/bridging is required for membrane-binding, and that both its chromosome-binding and membrane-binding activities are essential to its function.

© European Molecular Biology Organization

Noc also seems to recruit NBS-containing plasmids to the membrane in B.subtilis, and can also recruit the chromosome to the cell periphery when overexpressed to some undetermined level in E.coli. Interestingly, division is inhibited in both these cases, suggesting that drawing DNA close to the membrane can interfere with division in both organisms.

Based on these results, the authors propose a 'crowding' mechanism for Noc function, whereby the protein increases the density of nucleo-protein complexes near the membrane to a level sufficiently high to effectively interfere with assembly/stability of the division apparatus. More specific mechanisms are not excluded, however.

Overall, this study is an important contribution to the field, and the described properties of Noc will be of interest to a wider audience. However, parts of the study/presentation are incomplete in my opinion.

My critiques/comments are both of a technical (e.g. missing information on protein levels and integrity) and conceptual (especially point 14a-d, below) nature. This will require some additional work/documentation.

Comments:

1) CCCP sensitivity of Noc-YFP localzation.
I don't quite understand the effect of CCCP on Noc-YFP localzation, or the interpretation in the discussion (lines 345-347). If membrane-binding were delta-psi dependent (L 345), one would expect Noc-YFP to resemble delta10-Noc-YFP and localize to the nucleoid. In stead, the fusion is still in peripheral clusters that now no longer need be associated with the nucleoid. Why would CCCP affect DNA-binding of the full length Noc-YFP fusion?

When Noc is in association with the membrane the amphipathic helix is sensitive to changes in delta-psi. If delta-psi then collapses, *e.g.* upon CCCP treatment, then the binding of the amphipathic helix is altered, perhaps enhanced or stabilised, resulting in it being ‘pulled’ away from the DNA. A similar effect is seen with other delta-psi sensitive proteins *e.g.* MinD *etc.* (Strahl & Hamoen, 2010). But if Noc is not in contact with the membrane in the first place *e.g.* NocNΔ10 then this cannot happen. In the discussion we explore the possibility that delocalisation is due not only to the delta-psi sensitivity of the amphipathic helix but also to the increased fluidity of the membrane resulting from the altered function of another delta-psi sensitive protein, MreB (Strahl *et al*, 2014). To avoid any confusion we have replaced the term delta-psi dependent with the term delta-psi sensitive throughout the text and reworded the point about membrane fluidity.

2) Figure S1
The point of using the ATP synthase mutant (i.e. ATP-levels are less sensitive to CCCP) may escape

a good chunk of readers. The rationale with some references should be explained in the text or the figure legend.

As suggested, we have added a brief description of the rationale behind this experiment along with appropriate references to the legend of Fig. S1.

3) A key to how colour relates to percent identities in figures S2 and S7A is missing. Please include in legends.

A key to the colour scheme has now been added to Figure S2 and is also referred to in the legend to Fig. S7.

4) Figure S4A
a) The panel is confusing without an explanation in the legend that you are measuring cell length after OVERproduction of Noc.
b) The panel is also incomplete without the length distributions of wt and noc- cells for comparison.

We have now repeated this experiment with the inclusion of the requested control strains (*i.e.* 168 wt and DWA117 *noc*-). The results were similar to our previous experiments and the legend has been updated to clarify that the cell length in the other strains is being measured after Noc overproduction.

c) At what level of IPTG would Noc be expressed at normal level?

The $P_{spac(hy)}$ promoter used in these strains produces approximately WT levels of Noc without any induction due to 'leakiness' of the promoter. However, we previously established that in otherwise WT cells Noc must be overproduced to have an effect on cell length (Wu & Errington, 2004).

5) Figure S3 is incomplete as well.
a) What were the approximate MW's of Noc proteins?
b) How does this compare to the calculated MW's of monomer or dimer?
c) What were the MW standards, and were did they elute?

Due to resolution limitations of the preparative column used in our original analysis we have now repeated these experiments using an Analytical Gel Filtration column. As requested, a detailed description of the method has been added to the supplementary methods. The elution profiles and calibration curve/standards are shown in Fig. S3 and the approximate MW's of Noc proteins are stated in the legend and compared with the theoretical values. Our results indicate that Noc and NocNΔ10 proteins behave similarly by forming multimers (dimers and tetramers) whereas the NocCΔ50 protein behaves as a monomer.

© European Molecular Biology Organization

6) Table S1, functionality of mutants
a) Did authors do Westerns to ensure that mutants are not more (e.g. S4L) or less (all others) stable than wt?

Western blotting indicated that all proteins are produced a comparable levels. This is now stated in a footnote to Table S1 and refers the reader to the corresponding western blots, which are shown in Fig. S11.

b) What does 'More membrane' (S4L) mean?

This reflects the observation (shown in Fig. 2 J-K) that S4A-mYFP shows a stronger association with the membrane as compared with WT Noc-mYFP. To try and clarify this point we have changed it in the table to 'enhanced membrane'.

c) The Table also seems incomplete. For example, several mutants (delta-K2, K2A, delta-F5S6, R7A, K14A) used in sporulation assays (Fig.S4B) are not included. Why not? It just seems odd that some mutants are used for some, but not all assays for Noc functionality.
d) Line 166. Why refer to the sporulation assay here, which is derivative at best? According to Table S1 it seems as if K2 and R7 actually do contribute to Noc function.

Some of the mutants we made were not tested as YFP fusions because the ability to inhibit sporulation in the overproduction assay has consistently proven to be a reliable indicator of Noc functionality. We also make the distinction in the text that when the charges on residues K2 and R7 are inverted *i.e.* K2E, R7E they do affect function (Table S1) but that they do not seem to be necessary for function since the alanine mutants *i.e.* K2A, R7A and K14A all inhibit sporulation when overproduced (Fig. S4B).

As requested, we have now constructed and tested NocK14A-YFP. In line with its ability to inhibit sporulation when overproduced, the K14A-YFP fusion has a WT localisation pattern and rescues the growth defect of a *noc minCD* mutant. These results have been added to Table S1.

7) Figure 3
The composition of the figure suggests that the YFP fusion to HCV-Noc was not functional.
a) Please discuss if the fusion was functional or not.

As requested, we have now tested the functionality of the ^{HCV}AH-NocNΔ10-YFP fusion. As now described in the Results and shown in Fig. S5B, the fusion protein is functional and able to rescue the growth defect of a *noc minCD* mutant.

© European Molecular Biology Organization

b) What were growth conditions for panels G and H?

To aid the reader we now state the genotype of the strain and the growth conditions in the figure legend *i.e.* DWA193 (Δ*noc*, P_{xyl}-^{HCV}AH-*noc*NΔ10-*yfp*) grown at 30°C in CH medium.

8) Figure S5, TM-Noc
I'm somewhat surprised that the TM-Noc version does not seem to correct delta-Noc, delta-min at all.
a) Did you try this fusion at lower expression levels?

We repeated the experiments with a titration of the inducer (0.05, 0.125, 0.25, 0.5 and 1 % w/v xylose). However we saw no evidence that the TM-NocNΔ10 fusion can rescue the growth defect of a *noc minCD* mutant at any level of induction.

b) The results suggest the fusion should actually be toxic in wt or delta-noc cells at these levels of expression. Was it?

Strain DWA548 (Δ*noc*, P_{xyl}-TM-*noc*NΔ10-m*yfp*) can grow on plates with full induction without an obvious growth defect. However, as discussed in the Results the cells regularly exhibit chromosome segregation defects and have bisected chromosomes. Nevertheless, this phenotype was heterogeneous and many cells appear to contain relatively unperturbed chromosomes, which are now shown in Fig. S5D by the inclusion of an additional image. We also hypothesise in the discussion that this relatively mild effect may result from the protein being confined at the membrane and thus unable to freely access the DNA.

c) Does the TM-Noc fusion still not correct delta-Noc, delta-min at levels were it is not toxic to wt cells?

See points above.

d) One might expect chromosome bisection (as in panel C) to results in an SOS response. However, cells still seem to be dividing. Perhaps the authors can comment on this?

Indeed, one of the cells shown in the top right hand panel of Fig. S5D contains a fragmented chromosome and is clearly elongated without any obvious division. Other cells with damaged chromosomes that are still dividing may simply be in the early stages of activating the SOS response.

9) Figure S6

© European Molecular Biology Organization

A Western of the fusions that fail to accumulate on the membrane (panels B and C) needs to be included, to ensure the fusions are not simply degraded.

Western blots showing that the various intact fusion proteins are produced are now shown in Fig. S6 panels D and E and are referred to in the Results.

10) Figure 4, spreading mutants
a) Again, the correction and transdominant experiments would be more convincing with a Western showing that all Noc variants are present at about the same level.

Western blots showing that the various Noc proteins are all produced at comparable levels are now shown in Fig. S7C and are referred to in the main results.

b) I would expect the delta-10 mutant to become transdominant at some level of overexpression. What is the level of overexpression (versus WT) in the transdominant assays approximately?

This was also our initial expectation. Based on the blot shown in Fig. S7C it is clear that the proteins are overproduced at several times the native level. In the discussion we have interpreted this result as that the complexes of Noc don't have to be saturated with N-termini in order to function. However, other explanations may become apparent *e.g.* perhaps the excess NocNΔ10 molecules are somehow unable to disrupt the complexes associated with the membrane?

c) Line 239. Please indicate that these sporulation assays are not shown.

Done.

11) NBS plasmids
a) I assume that movies 4 and 5 are not TIRF microscopy? Wouldn't TIRF be more convincing if the point is to show more plasmid at the periphery of the cell?

Correct, these are epi-fluorescence movies. We did try TIRF with similar results. However, the mCherry channel on TIRF in this set up was very noisy and the images produced were rather poor. We therefore chose to present the epi-fluorescence movies since they provide the reader with the clearest images that are easy to interpret.

b) Line 284. I'm actually more struck by the impression that the NBS plasmid now seems to prefer co-localization with nucleoids in the presence of Noc (i.e. it seems relatively depleted from internucleoid gaps, Figure 5C). Can the authors explain/comment on this?

© European Molecular Biology Organization

We agree that in the presence of Noc the plasmids are no longer concentrated solely at the cell poles and the inter-nucleoid gaps as they are in its absence (Figure 5B/Movie 4). However, Figure 5C/Movie 5 clearly shows that they redistribute towards the cell periphery throughout the entire cell rather than just specifically over the nucleoid.

c) Fig5E suggests that the delta-10 protein may cause plasmids to aggregate at poles. Did you look at the plasmid in this context? This might be consistent with DNA-bridging by Noc?

As the referee suggests, this might represent a DNA-bridging activity by NocNΔ10 'trapping' plasmids. Equally though, since high-copy number plasmids, like the one carrying the NBS array, have a tendency to accumulate at the cell poles (Fig. 5B), and similar polar accumulation has recently been observed in *E. coli* (Reyes-Lamothe *et al*, 2014), this may simply reflect the binding of NocNΔ10 to plasmids that are already accumulated at the cell poles.

12) Expression in E.coli
a) It would be very helpful to know how much Noc protein is produced in these experiments, relative to say the normal level in B.subtilis. For example, a titration western with normalization to total protein or cell number could be added to figure S9.

We show in Figure S9 that the various Noc and hybrid proteins are overproduced at similar levels and state in the Results that we are using a high-copy number plasmid. Thus, the various proteins will undoubtedly be produced at much higher levels than in *B. subtilis*. The key control in our view is that even though the proteins are all overproduced at similar levels they have different activities and reflect the properties defined in *B. subtilis*. This strongly supports the interpretation that these effects are not due to some non-specific effect of protein overproduction.

b) Is Noc clustering/spreading still relevant at these levels of expression? Do the spreading mutants have any effect in E.coli?

Since our preliminary experiments indicated that producing the spreading mutants in *E. coli* does not recruit DNA to the membrane and the system lacks the NBSs we did not investigate these mutants further at this time.

c) Is the Noc-Spo hybrid functional in B.subtilis? I realize there are only few parS sites, but even if it has only partial 'Noc-like' activity, this would be of interest and might argue in favour of the model.

A good suggestion, we had the same thought. However, several formidable technical hurdles prevented this from being done. Foremost, the importance of the various other interactions Spo0J is involved in, including *parS*/SMC and Soj (ParA), which would complicate interpretation of any results obtained.

13) Plasmid construction
A better description of plasmids and details on their construction is needed.
For example, I'm not familiar with the PA1/04/03 promotor. Is this extra strong (see point 12, above)?

As requested, a detailed description of plasmids and their construction has been added to the supplementary methods including a description of the $P_{A1/O3/O4}$ promoter.

14) Model, figure 8
a) Does an NBS plasmid combined with fairly 'normal' levels of Noc cause division inhibition in E.coli as it does in B.subtilis? If so, you'd have a pretty good argument for the Noc-DNA 'crowding' model (though, see d below). If not, however, why not?

This is another good suggestion and we previously spent considerable time/effort trying to do this, with some encouraging preliminary results. Unfortunately, our attempts to interpret this type of experiment were hampered by problems with our NBS plasmids, since alone they appear to have some non-specific effect on *E. coli* that we as yet have not been able to understand.

b) Does a parS plasmid combined with fairly 'normal' levels of the Noc-Spo hybrid cause division inhibition in either B.subtilis or E.coli. Again, if so, it would argue for the crowding model, rather than anything specific to Noc.

This was not tested in *E. coli* but we did try in *B. subtilis* as described in the response to point 12C.

c) One conceptual problem I have with the crowding model is that the number of transertion-mediated contacts between the chromosome and the membrane is likely to be already so high in actively growing cells, that it is surprising if Noc-mediated contacts would make such a difference. I believe there are some numbers in the literature estimating the number of transertions at any one time. Maybe the authors can discuss this and the proposed contribution of Noc in a more quantitative fashion?

We agree that there is likely to be some distinction between the membrane-associated Noc complexes and the complexes generated by transertion. Nevertheless, since transertion itself remains relatively poorly understood and without detailed information on how Noc is binding to the membrane *e.g.* dwell-time, n^o of molecules present, amount of DNA recruited *etc.* it would in our opinion be premature to make a detailed quantitative comparison between the two processes. Indeed, we state in the final paragraph of the discussion that 'further work will be necessary to define the precise mechanism by which Noc acts'.

d) Lines 427-428. This 'natural ability' of the nucleoid to interfere with division may, of course, still depend on some other DNA-binding protein, which might interfere with the division apparatus in a specific way. The fact that such a factor has not yet been found in screens does not preclude this

© European Molecular Biology Organization

possibility. E.g. it could be DNA/RNA polymerase, or some other essential protein that can interfere with the division apparatus in an evolutionary conserved fashion, and relevant mutants may be difficult to generate/isolate. This may be worth pointing out to the reader.

We agree and discuss this idea in final paragraph wherein we state that the 'the nucleoid or else another associated factor(s)' may be involved.

15) Discussion, other points.
a) lines 361-362: Neither of the two statements is accurate, and they are also inherently inconsistent. If ParB-box mutants bind all over the chromosome, why wouldn't they bind plasmid DNA as well? The mutants may still prefer certain sequences on the chromosome, and whether or not they bind NBS plasmids was not really tested.

As described in the Results, since in contrast to other Noc proteins the ParB-box mutants are not excluded from the *ter* region of the chromosome, this provides good evidence that they bind throughout the entire chromosome. If so, and they are binding non-specifically to DNA, then the excess mass of chromosomal DNA over plasmid DNA (*c.a.* ≥10-fold) would favour preferential binding to the nucleoid. However, as the referee notes, it remains possible that these proteins may retain some sequence specificity for unknown sites on the chromosome. We have therefore rewritten these two statements to take account of this possibility.

b) Line 376. This statement does not logically follow the previous sentence. The TM-Noc fusion can most likely still bind DNA as it does cause segregation problems.

We have now expanded this sentence to reflect this fact but also to discuss our finding that the effects of the fusion are heterogeneous, as now shown in Fig. S5D.

c) Line 398. But, wouldn't the SOS response cause division inhibition due to chromosome breakage (see point 8)?

It seems reasonable that if the target of Noc were also proximal to the membrane they could most likely interact and thus inhibit division even in the absence of a chromosome damage response such as SOS. This, however, was not observed, and in many cells the chromosomes appear relatively normal and cells are dividing even though the TM-Noc-mYFP is clearly present in the membrane. This is now shown in Fig. S5D and, as discussed in response to point above, has been added to the discussion.

Referee #3:

This paper reports a comprehensive analysis of the Bacillus Noc, with surprising but convincing

results. Noc was previously identified as a factor that inhibits FtsZ-ring assembly over the nucleoid, but the mechanism was not known. Multiple searches have failed to find any direct interaction of Noc with FtsZ, or with downstream Z-ring proteins. Here Adams, Wu and Errington discovered that Noc has an N-terminal amphipathic helix that binds it weakly to the membrane. This binding is essential for its inhibitory activity. Also essential is the ability of Noc to spread from the single NBS, forming a multivalent complex that can bind to the membrane more tightly than a single Noc. These points are nicely demonstrated and discussed as a novel regulatory mechanism. An especially strong experiment was the reconstitution of the membrane binding and division block in E. coli. In the end I had expected to see some kind of mechanical model with road-blocks or barriers blocking advancing Z rings. I was pleased to see that the authors avoided this kind of detailed and premature speculation, and just left with the suggestion that the mass of nucleoid bound to the membrane somehow blocked the Z ring. A very nice study, important new discoveries and very well presented.

Minor editorial suggestion: Line 379 discusses the contrast of Noc mechanism to SlmA in E coli. This would be best to start as a new paragraph. Also, lines 388-392 would be better merged in that paragraph, leaving the new section to begin with "how does Noc inhibit.."

We agree and have implemented the change.

Explanatory References:

Reyes-Lamothe R, Tran T, Meas D, Lee L, Li AM, Sherratt DJ, Tolmasky ME (2014) High-copy bacterial plasmids diffuse in the nucleoid-free space, replicate stochastically and are randomly partitioned at cell division. *Nucleic Acids Res* **42:** 1042-1051

Strahl H, Bürmann F, Hamoen LW (2014) The actin homologue MreB organizes the bacterial cell membrane. *Nat Commun* **5:** 3442

Strahl H, Hamoen LW (2010) Membrane potential is important for bacterial cell division. *Proc Natl Acad Sci U S A* **107:** 12281-12286

Wu LJ, Errington J (2004) Coordination of cell division and chromosome segregation by a nucleoid occlusion protein in *Bacillus subtilis*. *Cell* **117:** 915-925

2nd Editorial Decision 05 December 2014

Thank you for the submission of a revised version of your manuscript. It has been evaluated by former referee #2, who now considers that your study is ready for publication (see below). I am pleased to inform you that your manuscript has been therefore accepted for publication in the EMBO Journal.

Referee #2:

The authors previously showed that the B.subtilis nucleoid occlusion (Noc) protein plays an important role in coupling assembly of the division apparatus at midcell to the location and

replication/segregation status of the chromosome. Noc binds multiple NBS sites on the chromosome and interferes with division, but how it does so is still enigmatic.

Here, the authors show that Noc also binds the membrane via a weak N-terminal amphipathic helix, that binding of Noc to DNA and subsequent spreading/bridging is required for membrane-binding, and that both its chromosome-binding and membrane-binding activities are essential to its function. Noc also seems to recruit NBS-containing plasmids to the membrane in B.subtilis, and can also recruit the chromosome to the cell periphery when overexpressed to some undetermined level in E.coli. Interestingly, division is inhibited in both these cases, suggesting that drawing DNA close to the membrane can interfere with division in both organisms.

Based on these results, the authors propose a 'crowding' mechanism for Noc function, whereby the protein increases the density of nucleo-protein complexes near the membrane to a level sufficiently high to effectively interfere with assembly/stability of the division apparatus. More specific mechanisms are not excluded, however.

Overall, this study is an interesting and important contribution to the field, and the described properties of Noc will be of interest to a wider audience.

The authors have responded effectively to my original comments, and the manuscript is now more complete and approachable.
